# NMR- and MS-based multiplex approach for comparative metabolome profiling of *Curcuma caesia versus curcuma longa* rhizomes and in relation to in vitro biological effects

**DOI:** 10.1038/s41598-025-30956-9

**Published:** 2025-12-18

**Authors:** Radwa H. El-Akad, Tarik A. Mohamed, Alexandru Nicolescu, Teshome Degfie Beshah, Sherif R. Abdel-All, Andrei Mocan, Mohamed A. Farag

**Affiliations:** 1 Pharmacognosy Department, Pharmaceutical and Drug Industries Research Institute, National Research Centre, P.O. Box 12622, Cairo, Egypt; 2 Chemistry of Medicinal Plants Department, National Research Centre, 33 El-Bohouth St., Dokki, 12622 Giza Egypt; 3 Laboratory of Chromatography, Institute of Advanced Horticulture Research of Transylvania, University of Agricultural Sciences and Veterinary Medicine, 3–5 M˘an˘aștur Street, Cluj-Napoca, 400372 Romania; 4 Department of Chemistry, Adama Science and Technology University, P.O. Box 1888, Adama, Ethiopia; 5 Institute of Pharmaceutical Sciences, Adama Science and Technology University, Adama, P.O. Box 1888 Ethiopia; 6 Department of Pharmacognosy, Faculty of Pharmacy, Alsalam University, Kafr Al Zayat, 31611 Al Gharbia Egypt; 7 Department of Pharmaceutical Botany, “Iuliu Hațieganu” University of Medicine and Pharmacy, Gheorghe Marinescu Street 23, Cluj-Napoca, 400337 Romania; 8 Pharmacognosy Department, College of Pharmacy, Cairo University, Cairo, 12613 Egypt

**Keywords:** Blue curcuma, C. caesia, C. longa, Metabolites profiling, UPLC/MS/MS, GC/MS, NMR, Biochemistry, Biological techniques, Chemistry, Drug discovery, Plant sciences

## Abstract

The study presents the first integrated comparative metabolomics approach of *Curcuma caesia* known as blue curcuma, that is relatively less explored, compared with the well-known yellow curcuma *Curcuma longa* using a multiplex analytical approach viz., UV, NMR, UPLC/MS/MS, GC/MS and SPME/GC/MS. The study targeted fingerprinting and chemical characterization of their primary and secondary metabolites as well as annotation of volatiles affecting their aroma and culinary uses. Datasets were further modelled using multivariate data analyses (PCA and OPLS) for samples’ classification and discrimination. The study revealed several newly reported metabolites in blue curcuma and assigned discriminating biomarkers for each species i.e. sesquiterpenes in blue curcuma vs. curcuminoids in yellow curcuma. Bioactivity assays, including antioxidant and antidiabetic activities, showed moderate activity for *C. caesia* relative to *C. longa*, while both species were inactive as anti-obesity agents.

**Supplementary Information:**

The online version contains supplementary material available at 10.1038/s41598-025-30956-9.

## Introduction

*Curcuma* is a prominent genus in the family Zingiberacea that comprises ~ 100 species; globally recognized for their applications in traditional medicine, culinary arts, food industry, therapeutic products, cosmetics, as well as sources for natural pigments^1,2^. Despite being native to tropical and subtropical areas in Asia, the word “*Curcuma”* is originally derived from the Arabic word “kurkum” which means “yellow” referring to the rhizome color^2,3^.

*Curcuma longa*, known as yellow turmeric, is the most notable species that has a long ethnobotanical history in Ayruvedic and Unani medicine and reported for wide array of pharmacological activities attributed to its enriched phytochemical reservoir viz. curcuminoids, aroma compounds, and polyphenols^2–4^. In contrast, *C. caesia*, known as blue/black turmeric after its distinguished rhizome color, remains relatively less explored despite being traditionally used for the treatment of various ailments including gastrointestinal tract disorders, wound healing, respiratory disorders, fever, rheumatoid arthritis, among several others^5,6^. Previous phytochemical studies reported sesquiterpenes, flavonoids and volatile constituents in blue curcuma as well as positively reacting to screening assays for alkaloids, tannins and phenolics^5,7–9^. Both species are reported to have significant pharmacological activity against cancer, inflammation, bacterial and fungal infections, liver diseases, and other metabolic disorders^2,3,5,6,8^.

Despite extensive studies on *C. longa* and increasing interest in *C. caesia*, an integrated and comprehensive comparative study between both species has yet to be performed that aids provide deeper insight on the metabolome of blue curcuma, in particular. This study presents the first multiplex analytical approach for a complementary metabolomics overview of both blue and yellow curcuma, using fingerprinting techniques i.e., ultraviolet absorbance (UV), nuclear magnetic resonance (NMR), versus hyphenated techniques i.e., ultra-performance liquid chromatography coupled to high resolution mass spectrometry (UPLC/HR-MS/MS), gas chromatography coupled to mass spectrometry (GC/MS) and solid phase microextraction-(SPME-GC/MS). The multi-platform metabolomic analyses presented in this study offer untargeted chemical characterization of polar, semi-polar and non-polar metabolites in addition to volatile constituents. Multivariate data analyses including principal component analysis (PCA) and orthogonal partial least squares discriminant analysis (OPLS-DA) were employed to classify among samples and biomarker assignment. Furthermore, antioxidant activity was investigated using different assays i.e. 1,1-diphenyl-2-picrylhydrazyl (DPPH), 2,2’-azino-bis(3-ethylbenzothiazoline) 6-sulfonic acid (ABTS) and ferric reducing antioxidant power (FRAP) to assess their free radical scavenging activity comparatively, alongside quantification of their total phenolic and flavonoid content. Further, in vitro assay of both turmeric samples against glucosidase and lipase enzymes was investigated as a marker for diabetes or obesity management.

## Materials and methods

### Plant material

*C. caesia* and *C. longa* were collected from Chongzuo, Guangxi Province, during the winter of 2023. Dr. Kai Wang authenticated rhizomes at the Chinese Academy of Sciences, Beijing. Rhizomes were lyophilized overnight and stored in sealed containers until further assays. Specimen was deposited at the herbarium of Faculty of Pharmacy-Cairo University, voucher no.: 1−12-23-F.

### UV fingerprinting of C. caesia and C. longa chloroform extracts

Three grams of each freeze-dried powdered *Curcuma* sample was macerated with 30 mL chloroform for 2 h, then centrifuged and filtered. An aliquot of 200 µL was used, prepared from four replicates in a 96-well plate for the Gen 5 Greener UV microplate reader (Gen 5, kitted with a 96-well quartz cell with 1 nm spectral resolution in the UV region). Aliquots of each curcuma extract (200 µL) were pipetted into the microplate wells (*n* = 4) of a Gen 5 Greener UV microplate quartz reader (BioTek Instruments, Inc., Winooski, VT, USA). The absorption spectra were recorded in the range of 200–500 nm ^10^.

### NMR fingerprinting of C. caesia and C. longa methanol extracts

Five milliliters of the methanol extract were aliquoted and subjected to drying under a nitrogen stream till complete dryness, and pellet was further resuspended in 700 ul CD_3_OD containing 0.96 mM hexamethyldisilane (HMDS) as an internal standard. All spectra were obtained using a Varian/Agilent VNMRS 600 NMR spectrometer, operating at proton and carbon NMR frequencies of 599.83 MHz and 150.84 MHz, respectively, with a 5-mm inverse detection cryoprobe. The^1^H NMR spectra were recorded with a spectral resolution of 0.26 Hz/point (Spectral Width, SW = 8389 Hz, acquisition size = 64 K complex points, zero-filled to a final Fourier transform (FT) size of 128 K). The parameters used were as follows: pulse width (pw) = 6 µs (90°), relaxation delay = 22.3 s, acquisition time = 2.7 s, and 120 scans^11,12^.

HMBC spectra were acquired with a bandwidth of 14 ppm in F2 (^1^H) and 235 ppm in F1 (^13^C), using two scans per 256 increments for F1 and 2516 complex data points in F2. The HMBC experiments were optimized for long-range couplings of 8 Hz, with a relaxation delay of 1 s and an acquisition time of 0.15 s, resulting in a total acquisition time of 22 min. To minimize degradation and artifact formation, all NMR spectra were recorded from samples prepared immediately before data acquisition, within a 6-h time frame. Spectra were automatically phase-corrected, with proton and carbon chemical shifts referenced to TSP at (δ1H = 0.0 ppm) and internal CD_3_OD (δ13C = 49.86 ppm), respectively^11,12^.

Additionally, 2D-NMR spectra, including^1^H single bond^1^H total correlation spectroscopy (TOCSY), ^1^Hsingle bond^13^C heteronuclear single-quantum coherence (HSQC), and^1^H single bond^13^C HMBC, were recorded for selected samples using standard CHEMPACK 7.1 pulse sequences (zTOCSY, gHSQCAD, and gHMBCAD) implemented in the Varian VNMRJ 4.2 A spectrometer software.

### NMR quantification

For metabolites quantification using NMR spectroscopy, the peak areas of selected proton signals belonging to the target compounds and the internal standard (TSP) were integrated manually for all samples^11^. The following equation was applied for absolute metabolite level calculations^13^:

m_T_ = M_T_ X (I_T_/I_St_) X (X_St_/X_T_) X C_St_ X V_St_.

m_T_: mass of the target compound [µg/g] in the solution used for^1^H NMR measurement.

M_T_: molecular weight of the target compound [g/mol].

I_T_: relative integral value of the^1^H NMR signal of the target compound.

I_St_: relative integral value of the^1^H NMR signal of the standard compound.

X_St_: number of protons belonging to the^1^H NMR signal of the standard compound.

X_T_: number of protons belonging to the^1^H NMR signal of the target compound.

C_St_: concentration of internal standard (TSP) in the solution used for^1^H NMR measurement [mmol/L].

V_St_: volume of solution used for^1^H NMR measurement [mL].

### Metabolites profiling of C. caesia and C. longa extracts via UPLC/HR-MS/MS

Briefly, 150 mg of each *Curcuma* fine powder specimen was homogenized with 5 mL MeOH (100% *v/v*) containing 10 µg/mL umbelliferone as an internal standard using an Ultra-Turrax mixer (IKA, Staufen, Germany) adjusted at 11,000 rpm, five times for 20 s periods, with intervals of 1 min between each mixing period to guard against temperature increases and heating effects. The resultant suspensions were then vortexed vigorously, centrifuged at 3000× *g* for 30 min, and filtered through a 22 μm pore size filter to remove plant debris. Then, 1 mL was aliquoted and filtered, and placed in LCMS vials^10^. Three independent replicates (*n* = 3) were done for each sample to assess biological variance.

The principal step of UPLC/HR-MS/MS analysis was conducted in triplicate (*n* = 3), with 2 µL introduced to an Dionex 3000 UPLC system (Thermo Fisher Scientific, Bremen, Germany) equipped with a HSS T3 column (100 × 1.0 mm, 1.8 μm; Waters^®^; column temperature: 40 °C), and a photodiode array detector (PDA, Thermo Fisher Scientific, Bremen). Binary gradient elution protocol at a flow rate of 150 µL/min using water/formic acid, 99.9/0.1 (*v/v*) (A) and acetonitrile/formic acid 99.9/0.1 (*v/v*) (B) varying between isocratic step for 1 min of 5% mobile phase B, then a linear increase of B from 5% to 100% over 11 min. The mobile phase was kept isocratic between 11 and 19 min at 100% B followed by decreasing towards 5% B within 1 min, and, finally, an additional 10 min, i.e., 20–30 min, for column re-equilibration using 5% B^10^.

The UPLC system was coupled with a high-resolution mass spectrometer using an Orbitrap Elite mass spectrometer (Thermo Fisher Scientific, Bremen, Germany) equipped with a HESI electrospray ion source (spray voltage: positive ion mode 4 kV, negative ion mode 3 kV; source heater temperature: 250 °C; capillary temperature: 300 °C; FTMS resolution: 30,000). Nitrogen was used as sheath and auxiliary gas. The CID mass spectra (buffer gas: helium; FTMS resolution: 15,000) were recorded in data-dependent acquisition mode (dda) using a normalized collision energy (NCE) of 35% and 45%^14^.

### GC/MS analysis of silylated primary metabolites in C. caesia and C. longa

Briefly, finely powdered samples (100 mg) were extracted with 5 mL 100% methanol, aided by sonication and for 30 min at room temperature using Branson CPX-952-518R (Branson Ultrasonics, Carouge, SA Switzerland). The extracts were then centrifuged (LC-04 C 80 − 2 C regen lab centrifuge, Zhejiang, China) at 12,000× g for 10 min. Three independent replicates (*n* = 3) were done for each sample to assess biological variance.

Then, 100 µL of the methanol extract was evaporated to dryness in opened screw-cap vials by using stream of nitrogen gas. For derivatization, 150 µL of N-methyl-N-(trimethylsilyl)-trifluoroacetamide (MSTFA) previously mixed (1:1) with anhydrous pyridine was added to the dried methanol extract and the mixture was incubated (Yamato Scientific DGS400 Oven, QTE TECHNOLOGIES, Hanoi, Vietnam) for 45 min at 60 °C. Silylated derivatives were separated on a Rtx-5MS Restek, Bellefonte, PA, USA (30-m length, 0.25-mm inner diameter and 0.25-m film)^14,15^..

### SPME-GC/MS analysis of volatile constituents in C. caesia and C. longa

Briefly, 20 mg of finely pulverized powder were placed in 1.5 ml SPME screw-cap vials quickly sealed and set on a tray that was kept at a constant 50 °C for 30 min. During that time, SPME fibers of stable flex coated with divinylbenzene/carboxen/polydimethylsiloxane (DVB/CAR/PDMS) 1 cm long of 50/30 µm purchased from Supelco^®^ (Oakville, ON, Canada) were used for sampling by incubation in the headspace above the sample. The analysis of volatile compounds was carried out on a Schimadzu^®^ GC-17 A gas chromatograph equipped with a DB-5 column (30 m x 0.25 mm i.d. x 0.25 μm film thickness; Supelco) and coupled to Schimadzu^®^ QP5050A mass spectrometer^14,15^.

### Multivariate data analyses

Both UPLC/MS/MS and GC-MS derived datasets for primary and secondary metabolites were subjected to unsupervised PCA and supervised OPLS-DA multivariate data analysis using SIMCA-P version 14.1 software package (Umetrics, Umeå, Sweden). All variables were mean-centered and scaled to Pareto variance. Unsupervised PCA was used to obtain a segregation pattern of the variance of metabolites among the samples. While the supervised OPLS-DA was used to verify PCA results, identify markers, and obtain detailed information on the distinctions among the studied specimens. The Chemometric models with several permutations were applied for models’ validation based on the main 2 indices, including R^2^ and Q^2^. Model predictability was indicated by Q^2^, and model goodness of fit was specified by R^15^.

### Quantification of total phenolic (TPC) and flavonoid contents (TFC)

The TPC and TFC in rhizome methanolic extract of *C. caesia* and *C. longa* were analyzed using 96-well plates with a SPECTROstar^®^ Nano Multi-Detection Microplate Reader (BMG Labtech, Ortenberg, Germany) following Folin-Ciocâlteu assay for TPC and aluminium chloride (AlCl_3_) colorimetric assay for TFC^16,17^.

In detail, for TPC determination: 20 µl extract (2.0 mg/mL) was mixed with 100 µL Folin-Ciocâlteu reagent, and 80 µL sodium carbonate (7.5% *w/v*) ad incubated for 30 min at room temperature followed by measuring the absorbance at 760 nm using gallic acid as reference drug (0.011–0.154 mg/mL, R^2^ = 0.99). Results are expressed as mean of triplicates (*n* = 3) ± S.D as gallic acid equivalent per gram of freeze-dried powder (GAE/g w).

For TFC determination: the absorbance of the mixture constituted of 40 µL extract (2.0 mg/mL), 40 µL sodium acetate (100 mg/mL) and 24 µL AlCl_3_ (25 mg/mL) in ethanol (total volume 200 µL) was measured at 430 nm (*n* = 3). Results were expressed as mean ± S.D. rutin equivalent per gram of freeze-dried powder (RE/g w). Calibration curve constructed of rutin at concentration range 0.025–0.125 mg/mL (R^2^ = 0.99).

### Bioactivity assays

#### Antioxidant activity determination^16^

##### DPPH assay

The assay was conducted following the same procedures described by Zayed et al.^16,18^,. Mixture of 30 µL extract and 270 µL 0.004% DPPH in methanol was incubated for 30 min. in darkness at room temperature followed by measuring its absorbance at 515 nm (*n* = 3). Results are expressed as mean ± S.D. mg trolox equivalent/gram weight of dried residue (mg TE/g w).

##### ABTS assay

The assay followed the exact procedures mentioned by Zayed et al.^16,18^,. The absorbance of the extract (2 mg/mL) and ABTS solution mixture was measured at 734 nm upon 30 min incubation at room temperature (*n* = 3). Results are expressed as mean ± S.D. mg trolox equivalent/gram weight of dried residue (mg TE/g w)^18,19^.

##### FRAP assay

The assay was performed as described by Zayed et al.^16,18^,. The absorbance of 20 µL extract (2 mg/mL) and 175 µL FRAP reagent mixture was measured at 593 nm upon 30 min. incubation at room temperature (*n* = 3). Results are expressed as mean ± S.D. mg trolox equivalent/gram weight of dried residue (mg TE/g w).

#### Antidiabetic activity determination via α-glucosidase Inhibition

The assay followed exact protocol in Zayed et al.^18,20^,. The mixture of extract (0.1–2 mg/mL), substrate, phosphate buffer, and yeast *α*-glucosidase was incubated at 37 °C before measuring the absorbance of the obtained color at 405 nm against blank and acarbose reference drug. % of Inhibition was calculated according to the following equation:

$${\%}\:\text{of}\:\text{Inhibition}=\frac{\:\text{Abs}_{(\text{control})}-\text{Abs}_{(\text{sample})}}{\text{Abs}_{(\text{control})}}\times100$$

The normalized logarithmic curve for % inhibition was used to express the data as IC_50_ ± SD (the concentration of the sample that could inhibit 50% of the enzyme for triplicates, *n* = 3).

#### Anti-obesity assay determination via lipase Inhibition

The assay was proceeded exactly as described by Zayed et al.^18^,. The crude porcine PL type II at 2.5 mg/mL (Sigma, EC 3.1.1.3) was suspended in 5 mM Tris–HCl buffer (pH 8.0, obtained using HCl). The mixture was centrifuged (6987.5×*g*, 8 min, 20.0 ± 1.0 °C), and the clear supernatant was isolated. Afterward, 40 µL of PL solution were pre-incubated for 15 min at 37 °C with 40 µL of varying concentrations (0.25–10 mg/mL) of each extract, then 4-nitrophenyl butyrate (*p*-NPB) substrate at a concentration of 10 mM in pure ethanol was added. The absorbance was measured spectrophotometrically at 405 nm in microplates, after another 5 min of incubation. Orlistat was used as a positive drug control for the assay^18,21^.

## Results and discussion

### UV fingerprinting of *C. caesia* and *C. longa* extracts

Considering the clear color difference in both rhizome pulps that is blue versus yellowish, UV fingerprinting was employed initially for identification of the nature of coloring matter in blue curcuma. Blue curcuma was investigated for the presence of azulenes (conjugated 5- and 7-membered fused ring system) that is known to impart bluish coloration. Due to its non-polar nature, and lower solubility in methanol, extraction was attempted with chloroform for both yellow and blue curcuma rhizome powder for comparative analysis (**Suppl. Fig. ****S1**). Extraction using methanol failed to show absorbance spectra for these phytochemicals. From literature, azulenes show maximum absorbance at λ_max_ 270–340 nm (< 350 nm) that corresponds to S₀ → S₂ transition (short wavelength)^22^, whereas, in this study blue curcuma showed an exclusive strong absorbance at longer wavelength ca. λ_max_ 400 nm, that inferred the presence of guaiazulenes (alkylated derivatives of azulenes)^23^. Guaiazulenes are lipophilic bicyclic sesquiterpene hydrocarbons having distinctive bluish-purple color and common in planta^23,24^. The alkyl substitution in guaiazulenes viz. methyl and isopropyl moieties; donates electrons to the conjugated ring π- system, thus, reduces the gap between the highest occupied molecular orbital (HOMO) and the lowest unoccupied molecular orbital (LUMO) i.e. ground and excited states, which in turn causes bathochromic shift in (S₀→S_1_) and (S₀→S₂) absorption^22,24–26^.

### NMR fingerprinting of *C. caesia* and *C. longa* methanol extracts

NMR analysis was performed on *C. caesia* and *C. longa* extracts to elucidate major metabolites differences and also aided in standardization for quality control. Leveraging prior NMR-based literature, peaks corresponding to major metabolites in *C. caesia* (BT) and *C. longa* (YT) were identified. As summarized in Table 1, a diverse array of metabolites was classified into three major phytochemical groups, comprising 11 chemically distinct compounds: 5 sesquiterpenes (1–5), 3 curcuminoids (6–8), and 3 primary metabolites (9–11). The identified metabolites are shown in Suppl. Fig. S2.

**Table 1:** Resonance assignments with chemical shifts of constituents identified in *Curcurma caesia* (blue curcuma) and *Curcurma longa* (yellow curcuma) *via* 600 MHz^1^H NMR, HSQC and HMBC spectra.

No	Metabolite	Assignment	^1^H (multiplicity)	HSQC	HMBC	Blue curcuma	Yellow curcuma
1. **Sesquiterpenes**							
**1**	Curdione	CH_3_−13	0.84 d (*J* = 6.6 Hz)	21.0	C-7 (55.5), C-11 (30.7), C-12 (20.0)	+	-
		CH_3_−12	0.94 d (*J* = 7.0 Hz)	20.0	C-7 (55.5), C-11 (30.7), C-13 (21.0)		
		CH_3_−14	0.94 d (*J* = 6.5 Hz)	18.8	C-3 (35.0), C-4 (47.0), C-5 (216.6)		
**2**	Xanthorrhizol	CH_3_−9	1.17 d (*J* = 3.0 Hz)	22.1	C-5 (146.5), C-8 (43.0) C-10 (34.5)	+	+
		CH_3_−14, 15	1.81 s	18.7	C-13 (134.2)		
		CH_3_−7	2.07 s	16.4	C-2 (123.2), C-3 (139.5)		
		H-8	2.48 m	39.8			
		H-12	5.34 m	129.1			
**3**	Furanodienone	H-15	1.26 s	14.0	C-9 (42.1), C-1 (132.2), C-10 (135.9)	+	-
		H-14	1.94 d (*J* = 1.2 Hz)	17.6	C-5 (133.2), C-4 (147.7) C-3 (41.0)		
		H-13	2.06 s	8.1	C-11 (123.2), C-12 (139.5)		
		H-1	5.16 dd (*J* = 5.0, 11.5 Hz)	130.5			
		H-5	5.87 s	131.8	C-6 (192.0), C-3 (41.0), C-14 (17.6)		
**4**	Curcuzederone	H-13	2.06 s	8.2	C-11 (123.2), C-12 (139.5)	+	+
		H-5	3.74 s	55.1			
		H-12	7.16 s	139.5	C-11 (123.2), C-8 (159.0)		
**5**	Curcuminol G	H-15	1.09 s	18.5		+	+
		H-14	1.83 s	18.7			
		H-13	1.87 s	10.5	C-11 (136.0), C-12 (180.0)		
2. **Curcuminoids**							
**6**	Curcumin I	H-4,4`	7.55 d (*J* = 15.8 Hz)			-	+
		H-6,6`	7.20 d (*J* = 1.7 Hz)				
		H-10, 10`	7.09 dd (*J* = 8.6, 1.7 Hz)				
		H-3,3`	6.59 d (*J* = 15.8 Hz)				
**7**	Curcumin II	H-4,4`	7.55 d (*J* = 15.8 Hz)			-	+
		H-6	7.20 d (*J* = 1.7 Hz)				
		H-6`, 10`	7.47 d (*J* = 8.6 Hz)				
		H-7`, 9`	6.80 d (*J* = 8.6 Hz)				
		H-3,3`	6.59 d (*J* = 15.8 Hz)				
**8**	Curcumin III	H-4,4`	7.55 d (*J* = 15.8 Hz)			-	+
		H-6`, 10`	7.47 d (*J* = 8.6 Hz)				
		H-6, 10	7.47 d (*J* = 8.6 Hz)				
		H-7`, 9`, 7, 9	6.79 d (*J* = 8.6 Hz)				
		H-3,3`	6.59 d (*J* = 15.8 Hz)				
**3. Primary Metabolites**							
**9**	Methionine	S-CH_3_	2.07 s	17.5	CH_2_ (29.9)	+	+
		NH_2_-CH	3.30 m	48.5			
**10**	Choline	N(CH_3_)_3_	3.18 s	47.5		+	+
**11**	Fumaric acid	CH = CH	6.64 br s	134.2		+	+

#### Sesquiterpenes

**Fig. 1:**
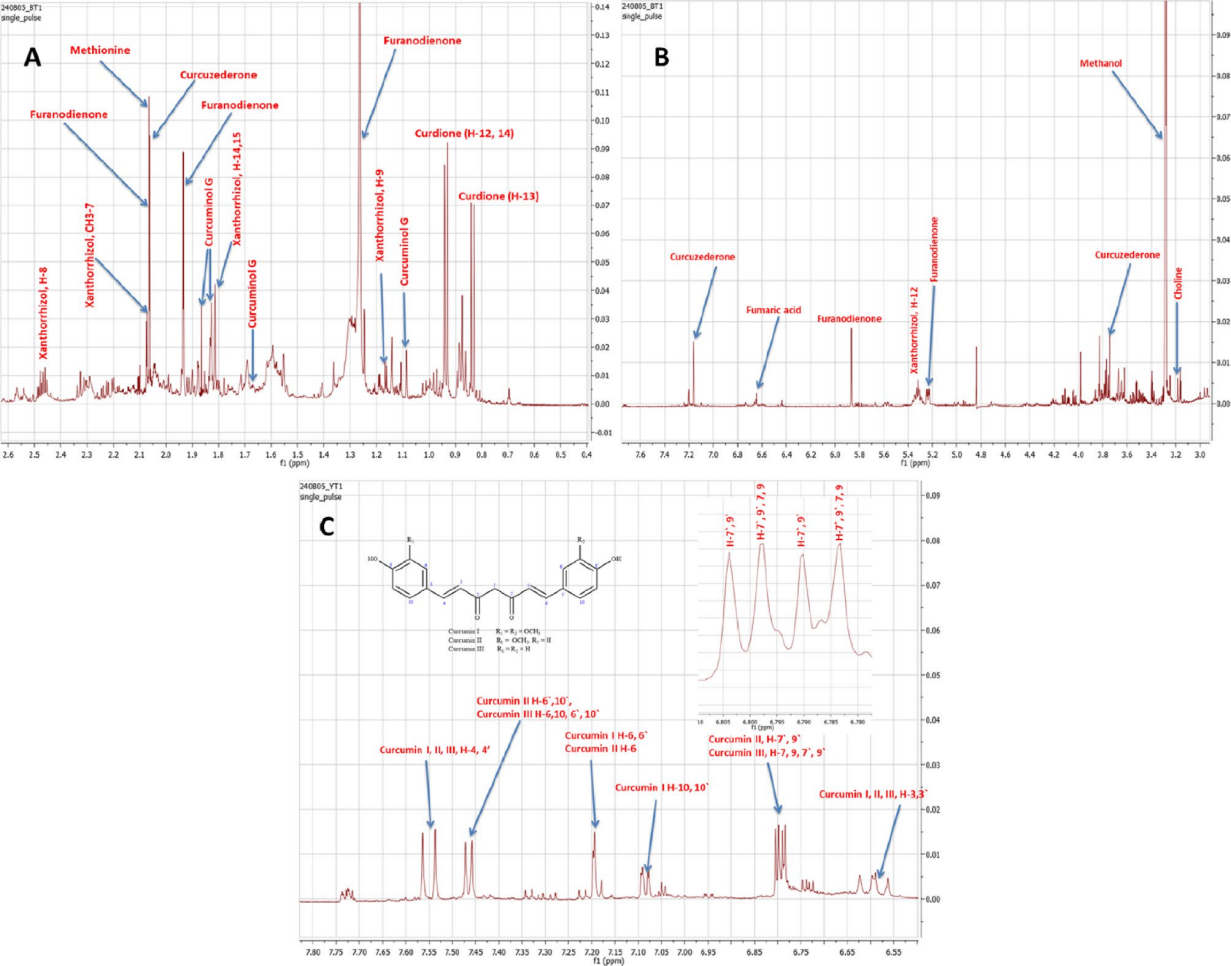
^1^H-NMR spectra (CD_3_OD, 500 MHz) of *Curcuma caesia* (A, B: δ_H_: 0–8 ppm) and *Curcuma longa* (C, δ_H_: 6.5–8 ppm) used in the curent study. The spectra are labelled with maor key metabolites, while peaks assignments are summerized in Table 1.

Sesquiterpenes (15-carbon terpenoids) represent a structurally diverse class of secondary metabolites. Curdione, exclusive to *C. caesia*, displays three methyl doublets (CH₃−13 at δ_H_ 0.84, *J* = 6.6 Hz; CH₃−12 and CH₃−14 at δ δ_H_ 0.94), confirming its germacrane skeleton. Key HMBC correlations from CH₃−14 to the ketone C-5 (δ_C_ 216.6) validate ring fusion^27,28^. Xanthorrhizol, present in both species, exhibits a trisubstituted olefin (H-12, δ_H_ 5.34) and characteristic methyl signals: a doublet (CH₃−9, δ_H_ 1.17, *J* = 3.0 Hz) and singlets (allylic CH₃−14/15, δ_H_ 1.81; vinylic CH₃−7, δ_H_ 2.07). HMBC correlations link CH₃−7 to olefinic C-2 (δ_C_ 123.2) and C-3 (δ_C_ 139.5), confirming its bisabolane core^29,30^. Furanodienone (both species) features a furan ring evidenced by H-5 (δ_H_ 5.87, s) and methyl groups (H-13, δ_H_ 2.06; H-14, δ_H_ 1.94, d, *J* = 1.2 Hz), with HMBC correlating H-14 to furan carbons C-3 (δ_C_ 41.0), C-4 (δ_C_ 147.7) and C-5 (δ_C_ 133.2) (Oon et al., 2016). Curcuzederone (both species) showed a phenolic structure with an aromatic proton (H-12, δ_H_ 7.16, s) and methoxy group (H-5, δ_H_ 3.74, s). Curcuminol G, exclusive to *C. caesia*, is identified by three methyl singlets (H-13–H-15, δ_H_ 1.09–1.87) and an HMBC correlation from H-13 to a carbonyl (C-12, δ_C_ 180.0), suggesting a sesquiterpenoid acid/ester (Fig. 1A and B, **Suppl. Fig. S3 and S4**, Table 1**)**^27–32^.

#### Curcuminoids

Curcuminoids, diarylheptanoid pigments exclusive to *C. longa*, share diagnostic *trans*-olefinic protons: H-3/H-3′ (δ_H_ 6.59, d, *J* = 15.8 Hz) and H-4/H-4′ (δ_H_ 7.55, d, *J* = 15.8 Hz), confirming the conjugated diene system (Khattab et al., 2022). Curcumin I displays asymmetric *meta*-substituted aromatics: H-6/H-6′ (δ_H_ 7.20, d, *J* = 1.7 Hz) and H-10/H-10′ (δ_H_ 7.09, dd, *J* = 8.6, 1.7 Hz). Curcumin II shows mixed substitution: one ring with *meta*-coupling (H-6, δ_H_ 7.20) and the other with *ortho*-coupling (H-6′/H-10′, δ_H_ 7.47, d, *J* = 8.6 Hz; H-7′/H-9′, δ_H_ 6.80, d, *J* = 8.6 Hz). Curcumin III exhibits symmetric *ortho*-substitution: equivalent aromatic protons for both rings (H-6/H-10 & H-6′/H-10′ at δ_H_ 7.47; H-7/H-9 & H-7′/H-9′ at δ_H_ 6.79). Their absence in *C. caesia* explains its lack of yellow pigmentation (Fig. 1C; Table 1**)**^27,33,34^.

#### Primary metabolites

Primary metabolites, conserved in both species, reflect essential cellular functions, and moreover nutritive value in both rhizomes. Methionine was identified by its sulfur-methyl group (S-CH₃, δ_H_ 2.07, s) and α-methine proton (NH₂-CH, δ_H_ 3.30, m), with HMBC correlating S-CH₃ to β-methylene (δ_C_ 29.9) (Wang et al., 2015). Choline showed a sharp singlet for its trimethylammonium group [N(CH₃)₃, δ 3.18, s]. Fumaric acid exhibits a broad singlet (δ_H_ 6.64) for its equivalent olefinic protons (CH = CH), consistent with its symmetric *trans*-structure. These compounds underpin universal metabolic roles: methionine in methylation, choline in membrane integrity, and fumaric acid in the Krebs cycle (Fig. 1A and B; Table 1**)**^27,30,31^.

### Quantitative^1^H-NMR analysis of key metabolites in *C. caesia* (BT), and *C. longa* (YT)

^1^H-NMR (qNMR) was employed to determine the absolute concentrations (µg/g) of key metabolites (7 metabolites out of 11 identified metabolites) underscores profound metabolic divergence between *C. caesia* (BT) and *C. longa* (YT), revealing species-specific biochemical strategies (Suppl.Table**S1****)** BT’s exclusive production of curdione (1.1 µg/g, germacrane-type) and furanodienone (1.4 µg/g, furanosesquiterpenoid) aligns with its traditional use in anti-inflammatory therapies, while the absence of these in YT highlights evolutionary pathway specialization^35^. Conversely, xanthorrhizol is more abundant in BT (1.5 µg/g) than in YT (1.4 µg/g; bisabolane-type), whereas YT’s exclusive accumulation of curcumin I (0.93 µg/g) directly correlates with its antioxidant and iconic pigmentation absent in BT due to silenced diarylheptanoid biosynthesis (Jantan et al., 2012). Notably, Curcuzederone exhibits preferential accumulation in BT (0.9 µg/g vs. 0.3 µg/g in YT), suggesting differential regulation of phenolic sesquiterpenoid pathways, whereas near-identical curcuminol G levels (0.3 µg/g BT vs. 0.1 µg/g YT) imply conserved enzymatic machinery for this sesquiterpenoid acid. The exclusive detection of choline in BT (0.1 µg/g) further indicates distinct primary metabolism, potentially supporting membrane biosynthesis in stress adaptation. These quantified disparities rooted in proton-specific integrals (e.g., 3 H for methyl groups vs. 1H for olefinics) provide not only chemotaxonomic markers for species authentication but also a biochemical rationale for their ethnopharmacological differentiation, with BT as a sesquiterpene-rich analgesic, and YT as a curcuminoid-centric antioxidant^36–38^.

### Metabolites profiling of *C. caesia* (BT) and *C. longa* (YT) rhizome methanol extracts *via* UPLC/HR-MS/MS analysis in both ionization modes

**Fig. 2:**
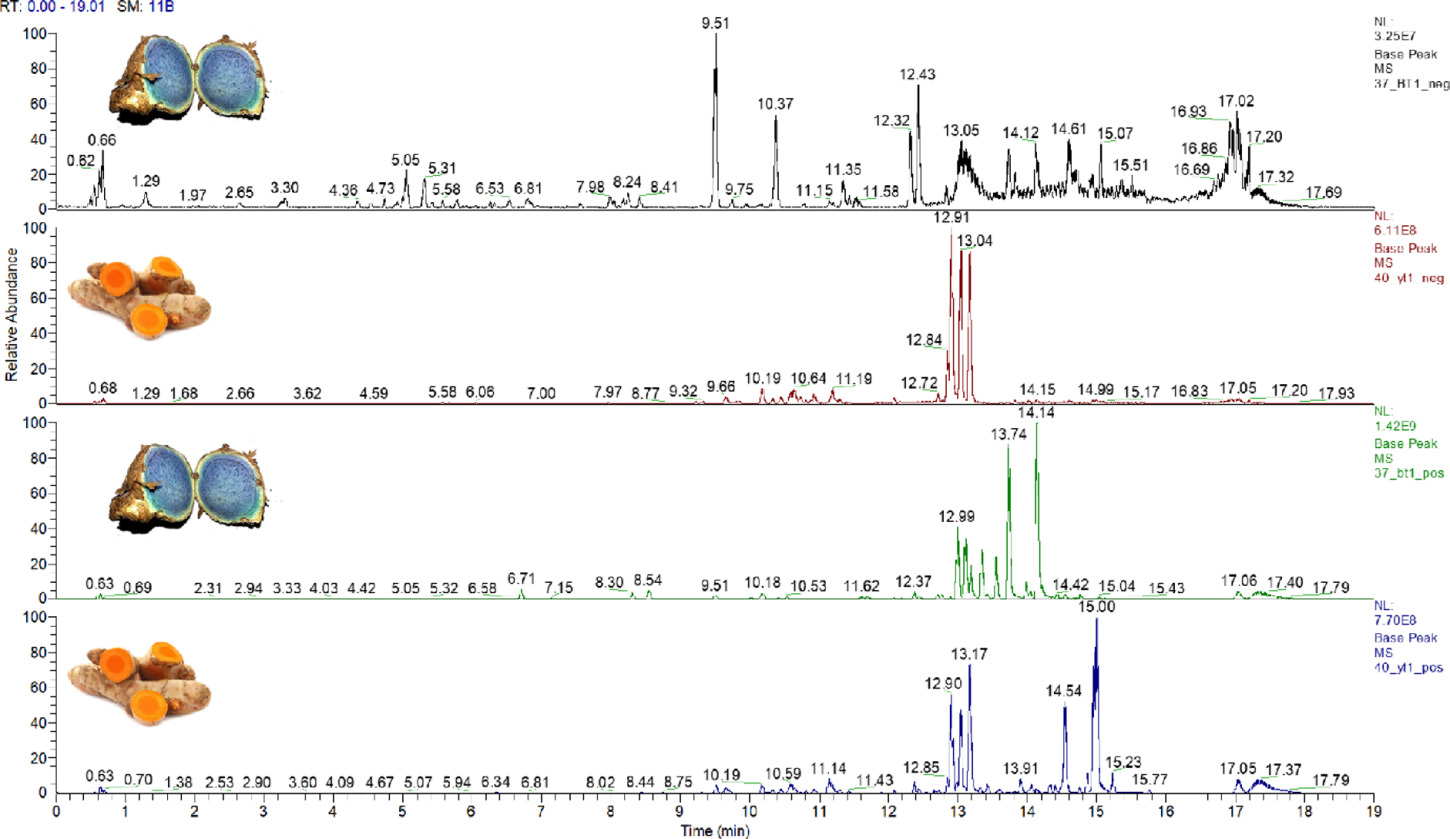
Base peak chromatograms of *C. Caesia* (blue curcuma) and *C. longa* (yellow curcuma) crude methanol extracts analyzed *via* UPLC/HR-MS/MS in both negative and positive modes.

**Fig. 3:**
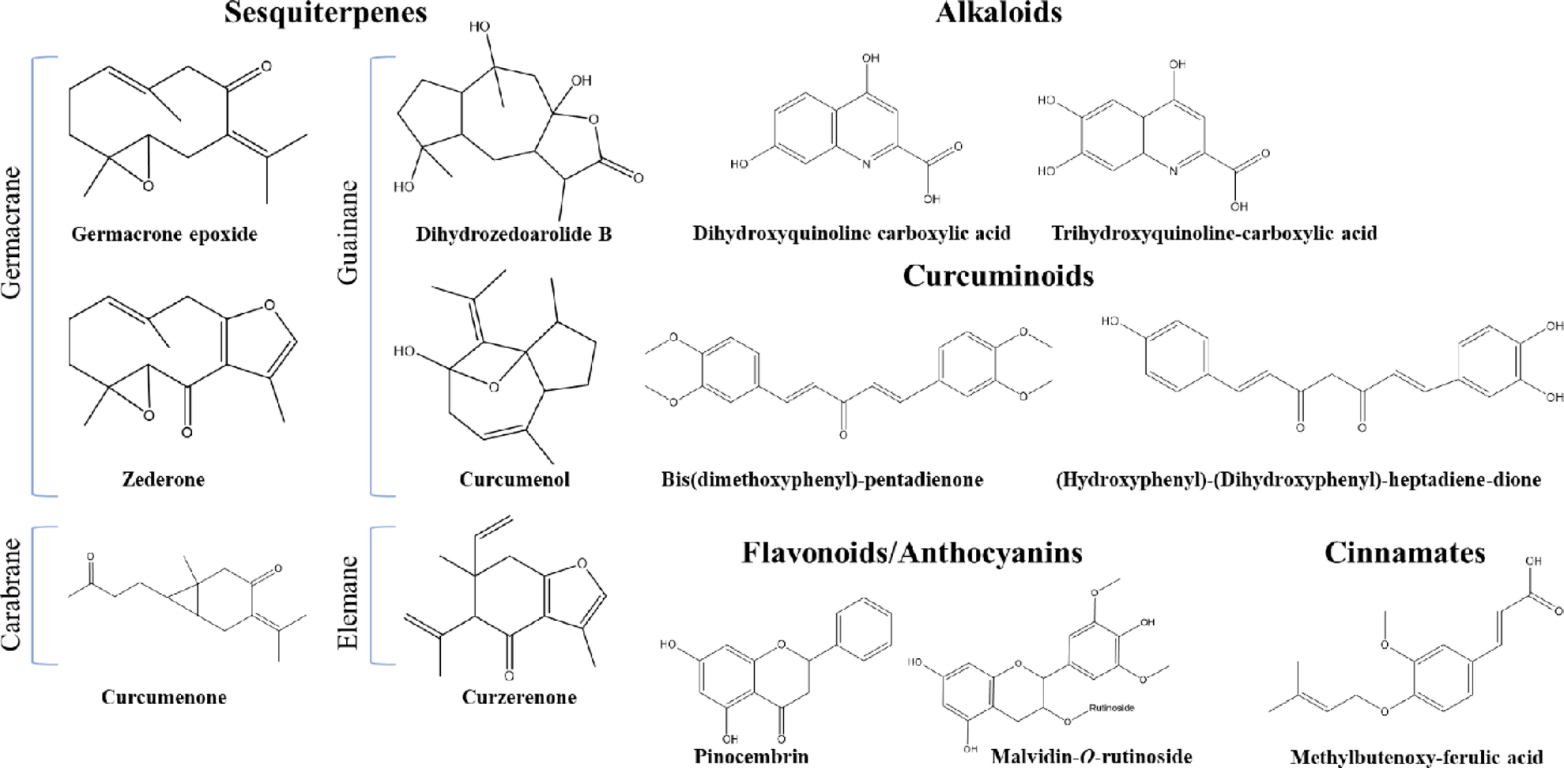
Structures of selected metabolites tentatively identified *via* UPLC/HR-MS/MS analysis in *C. caesia* and *C. longa* extracts as shown in Table 1.

Metabolites profiling of BT and YT *via* UPLC/HR-MS/MS analysis in both negative and positive modes provided improved secondary metabolites coverage for less abundant secondary metabolites not detected using NMR^39^. Gradient system of water (0.1% formic acid): acetonitrile eluted metabolites from the most to the least polar throughout a 20 min. runtime. Annotation of metabolites was based on MS data, including molecular ion, product ions, respective elemental formulae (error < 5 ppm), and fragmentation pattern; in accordance with previously published literature and databases e.g., Reaxys, HMDB, FooDB, Pubchem, and phytochemical dictionary of natural products, in addition to predicted spectra generated by CFM-ID (https://cfmid.wishartlab.com). Base peak chromatograms (BPC) of *Curcuma* sp. in both modes are shown in Fig. 2. The identified metabolites are listed in Table 2 based on their classes. Structures of selected metabolites annotated in both species are demonstrated in Fig. 3.

**Table 2:** Metabolites profiling of *Curcuma caesia* (blue curcuma) and *Curcuma longa* (yellow curcuma) rhizome methanol extracts *via* UPLC/HR-MS/MS analysis in both negative and positive ionization modes.

Peak	Rt (min.)	Identification	Molecular ion [M-H]^−^/[M + H]^+^	Elemental composition	Error (ppm)	Fragments MS^*n*^ ion	Blue Curcuma	Yellow Curcuma
**I- Sugars**								
1	0.59	Hexose	179.0547	C_6_H_11_O_6_^−^	1.6	161, 135, 99, 87, 71, 59	(+)	(+)
2	0.66	Lactobionic acid	357.1030	C_12_H_21_O_12_^−^	0.7	339, 177, 119, 101, 89, 87, 71, 59	(+)	(+)
3	0.68	Dihexosyl-*O*-glycerol	415.1444	C_15_H_27_O_13_^−^	0.4	341, 235, 179, 161, 101, 89, 71	(+)	
4	0.69	Trihexoside	503.1614	C_18_H_31_O_16_^−^	1.5	341, 179, 161	(+)	
5	0.7	Dihexoside	341.1082	C_12_H_21_O_11_^−^	1.1	179, 161, 101, 89, 71	(+)	(+)
6	0.71	Sugar derivative	267.0712	C_9_H_15_O_9_^−^	0.8	249, 237, 219, 150, 125, 113, 101, 89, 71, 59	(+)	(+)
7	1	Dipentoside	281.0875	C_10_ H_17_ O_9_^−^	2	113, 96, 87, 85, 71, 59	(+)	
**II- Organic acids**								
8	0.9	Malic acid	133.0128	C_4_H_5_O_5_^−^	1.9	115, 71	(+)	(+)
9	0.96	Citric acid	191.0188	C_6_H_7_O_7_^−^	0.9	173, 129, 111, 103, 87	(+)	(+)
10	1.58	Succinic acid	117.0179	C_4_H_5_O_4_^−^	2.6	99, 73, 55	(+)	
11	1.7	Benzoic acid	121.0280	C_7_H_5_O_2_^−^	2.5	108, 101, 93, 91	(+)	
**III- Phenolics**								
12	1.6	Salicylic acid	137.0229	C_7_H_5_O_3_^−^	2	101, 93, 87, 66	(+)	
13	3.1	Gallic acid-*O*-hexoside	331.0663	C_13_H_15_O_10_^−^	1.1	315, 168, 150, 125, 89	(+)	
14	3.2	Hydroxy-methoxyphenyl-*O*-hexoside	301.0920	C_13_H_17_O_8_^−^	0.8	138, 123, 97	(+)	
15	3.53	Protocatechuic acid-*O*-hexoside	315.0715	C_13_H_15_O_9_^−^	1.1	153, 135, 108, 91	(+)	
16	3.7	Hydroxy-dimethoxyphenyl-*O*-hexoside = (Dimethoxy-hydroquinone-*O*-hexoside) = Leonuriside A	331.1027	C_14_H_19_O_9_^−^	0.4	168, 153, 138, 125, 97, 93, 83, 71, 59	(+)	
17	3.72	Protocatechuic acid	153.0183	C_7_H_5_O_4_^−^	0.4	135, 108, 91	(+)	(+)
18	4	Salicin	285.0973	C_13_H_17_O_7_^−^	1.5	267, 195, 177, 123	(+)	
19	4.06	Hydroxyphenyl propanoic acid-*O*-hexoside	343.1028	C_15_H_19_O_9_^−^	1.3	181, 163, 137, 119, 101, 89, 71	(+)	
20	4.08	Syringic acid-*O*-hexoside	359.0977	C_15_H_19_O_10_^−^	1.3	197, 182, 167, 153, 138, 123, 71	(+)	(+)
21	4.7	Eugenol-O-hexoside	325.1284	C_16_H_21_O_7_^−^	0.7	163	(+)	(+)
**IV- Flavonoids/anthocyanins**								
22	4.6	Epi/Catechin	289.0710	C_15_H_13_O_6_^−^	1.4	245, 205, 151	(+)	(+)
23	5.65	Malvidin-*O*-rutinoside	639.1912	C_29_H_35_O_16_^+^	1	493, 331, 150	(+)	
24	5.7	Quercetin-*O*-dirhamnoside	593.1498	C_27_H_29_O_15_^−^	0.4	300		(+)
25	6.88	Di-*O*-methyl-epi/catechin-*O*-hexoside	479.1552	C_23_H_27_O_11_^−^	0.48	317, 285, 255, 151, 131	(+)	
26	10.3	Naringenin	271.0606	C_15_H_11_O_5_^−^	1.6	253, 177, 151	(+)	
27	12.7	Pinocembrin	255.0654	C_15_H_11_O_4_^−^	1.07	213, 151		(+)
**V- Cinnamates**								
28	3.95	Hydroxy-ferulic acid-*O*-hexoside	373.1131	C_16_H_21_O_10_^−^	0.5	211, 193, 165, 149, 123, 105, 73	(+)	
29	4.85	Caffeic acid	179.0335	C_9_H_7_O_4_^−^	2	135	(+)	(+)
30	7.3	Cinnamic acid	147.0437	C_9_H_7_O_2_^−^	1	129, 103, 91, 77		(+)
31	8.2	Feruloyloxy-methoxycinnamic acid	339.0862	C_19_H_15_O_6_^−^	0.1	193		(+)
32	13.35	Methylbutenoxy ferulic acid	261.1124	C_15_H_17_O_4_^−^	1.1	217, 193, 189, 149, 137, 121, 105, 81, 79, 67	(+)	
**VI-Alkaloids**								
33	3.72	Dihydroxyquinoline carboxylic acid-*O*-hexoside	366.0824	C_16_H_16_NO_9_^−^	0.1	204, 185, 173, 160, 130, 107, 102, 93, 87, 73, 68	(+)	(+)
34	4	Trihydroxyquinoline-2-carboxylic acid-*O*-hexoside	382.0771	C_16_H_16_NO_10_^−^	0.6	366, 351, 220, 176, 121	(+)	(+)
**VII- Di/Aryl alkanoids (Curcuminoids)**								
35	9.5	Dihydroxy-(hydroxy-dimethoxyphenyl)-(hydroxy-methoxyphenyl) heptane	405.1879	C_22_H_29_O_7_^−^	0.02	390, 349, 192, 179, 165, 151, 135, 125, 109	(+)	(+)
36	9.83	Bis(hydroxyphenyl)-hydroxyheptenone	313.1437	C_19_H_21_O_4_^+^	1	207, 189, 163, 149, 119		(+)
37	9.86	Calebin A	385.1274	C_21_H_21_O_7_^+^	1.9	193, 191, 177, 161, 149, 145, 133, 97		(+)
38	9.9	(Hydroxyphenyl)-(Dihydroxyphenyl)-heptadiene-dione	323.0916	C_19_H_15_O_5_^−^	0.6	217, 189, 174, 161, 146, 133, 119, 109, 59		(+)
39	10.32	Curcumalongin A	353.1015	C_20_H_17_O_6_^+^	1.5	338, 321, 293, 269, 171, 153, 147		(+)
40	10.35	Hexahydrocurcumin	373.1647	C_21_H_25_O_6_^−^	0.5	193, 179, 165, 121	(+)	
41	10.48	(Dihydroxybenzylidene)-(hydroxy-methoxystyryl) furanone	353.1016	C_20_H_17_O_6_^+^	0.8	338, 337, 177, 153, 150, 123		(+)
42	10.6	Curcumalongin B	383.1117	C_21_H_19_O_7_^+^	1.8	368, 350, 294, 177, 153, 145, 121		(+)
43	10.7	Curcumalongin C	385.1274	C_21_H_21_O_7_^+^	1.8	269, 219, 195, 193, 177, 161, 153, 145, 133, 117		(+)
44	10.91	(Hydroxy-methoxyphenyl)-(hydroxyphenyl)-hydroxyheptadieneone	339.1230	C_20_H_19_O_5_^−^	1	219, 189, 175, 161, 149, 134, 119, 69		(+)
45	11.45	Curcolone	245.1174	C_15_H_17_O_3_^−^	1	227, 203, 161	(+)	(+)
46	11.48	Tetrahydrodemethoxycurcumin	341.1384	C_20_H_21_O_5_^−^	0.4	235, 205, 193, 179, 163, 135, 99	(+)	(+)
47	11.5	Tetrahydrocurcumin	371.1491	C_21_H_23_O_6_^−^	0.5	235, 193, 178, 135, 99	(+)	(+)
48	11.7	(Hydroxy-methoxybenzylidene)-(hydroxystyryl) furanone	337.1073	C_20_H_17_O_5_^+^	1	321, 305, 160, 147, 137, 123		(+)
49	12.4	Bis(hydroxy-methoxyphenyl)-heptatrien-3-one	353.1378	C_21_H_21_O_5_^+^	1.4	285, 259, 225, 197, 177, 161, 145, 137		(+)
50	12.84	Dihydrobisdemethoxycurcumin	309.1124	C_19_H_17_O_4_^−^	0.85	203, 189, 161, 145, 119, 93		(+)
51	12.88	Curcumin III, (Bisdemethoxycurcumin)	309.1116	C_19_H_17_O_4_^−^	1.9	189, 147, 131, 119		(+)
52	12.95	Bis(dimethoxyphenyl)-pentadienone	355.1532	C_21_H_23_O_5_^+^	2	265, 219, 201, 179, 177, 163, 145, 137	(+)	
53	13.05	Curcumin II (Demethoxycurumin)	339.1221	C_20_H_19_O_5_^+^	1.7	269, 255, 223, 195, 177, 147, 119	(+)	(+)
54	13.1	Curcumin I	369.1326	C_21_H_21_O_6_^+^	0.4	217, 177, 161, 145, 137, 117	(+)	(+)
55	13.25	Methoxycurcumin	397.1281	C_22_H_21_O_7_^−^	0.9	247, 217, 203, 179, 173, 158, 149, 134		(+)
56	14.53	ar-Turmerone	217.1579	C_15_H_21_O^+^	3	119, 103, 91		(+)
57	14.9	Dihydro-ar-Turmerone	219.174	C_15_H_23_O^+^	2.5	121, 105, 93, 83, 79, 55		(+)
**VIII- Sesquiterpenes**								
58	3.8	Dihydro zedoarolide B-*O*-hexoside	445.2061	C_21_H_33_O_10_^−^	0.6	283, 265, 247, 239, 221, 219, 203, 185, 167, 161, 151, 133, 111, 71	(+)	
59	4.16	Trihydroxy-guaianenone	429.2141	C_21_H_33_O_9_^−^	1.04	411, 393, 267, 249, 231, 179, 161, 142, 139, 119, 89, 71	(+)	
60	4.3	Dihydro derivative of Zedoarolide A	297.1331	C_15_H_21_O_6_^−^	0.1	279, 261, 235, 207, 199, 165, 127, 83	(+)	
61	4.4	Zedoarolide B-*O*-hexoside	443.1911	C_21_H_31_O_10_^−^	0.03	281, 263, 249, 237, 223, 219, 179, 161, 89, 71	(+)	
62	4.8	Zedoalactone A/C/E-*O*-hexoside	427.1966	C_21_H_31_O_9_^−^	0.1	265, 247, 235, 207, 189, 161	(+)	
63	5.02	Zedoarolide A	295.1178	C_15_H_19_O_6_^−^	0.7	277, 259, 233, 215, 153, 125	(+)	
64	5.05	Hydroxy-(hydroxymethyl)-dimethyl-tetrahydroazulenofuranone (wenyujinin F)	261.1126	C_15_H_17_O_4_^−^	1.8	219, 217, 201, 199, 191, 175, 163, 159, 135, 123, 109, 95, 85, 65, 57	(+)	
65	5.07	Zedoalactone B/D	279.1229	C_15_H_19_O_5_^−^	0.7	261, 243, 235, 217, 199, 177, 163, 153, 137, 109, 95	(+)	(+)
66	5.8	Zedoarolide B	281.1387	C_15_H_21_O_5_^−^	1.4	263, 250, 237, 219, 207, 201, 189, 171, 139, 123, 83	(+)	(+)
67	6.9	Aerugidiol-*O*-hexoside	411.2014	C_21_H_31_O_8_^−^	0.1	249, 231, 213, 191, 179, 161,	(+)	
68	7.1	Unknown sesquiterpene lactone	277.1074	C_15_H_17_O_5_^−^	1.4	247, 233, 203, 185, 177, 161, 145, 133	(+)	
69	8	Dihydro derivative of Zedoarolide B	283.1543	C_15_H_23_O_5_^−^	1.05	265, 239, 221, 219, 203, 185, 167, 151, 133	(+)	
70	11.3	Hexahydro-dihydroxy-trimethylazulenofuranone (wenyujinolides A/B)	263.1281	C_15_H_19_O_4_^−^	1.4	245, 201, 219, 191, 149,137, 125, 109, 97	(+)	
71	12.5	Zedoalactone A/C/E	265.1452	C_15_H_21_O_4_^−^	0.2	221, 191, 147	(+)	
72	12.9	Germacrone epoxide	235.1685	C_15_H_23_O_2_^+^	2	217, 207, 193, 189, 175, 161, 147, 135, 119, 107, 105, 93, 79, 69, 67	(+)	
73	12.97	Furandiene	217.1585	C_15_H_21_O^+^	0.8	199, 189, 175, 161, 135, 119, 107, 105, 93, 79, 69	(+)	
74	13	Confertin	247.1330	C_15_H_19_O_3_^−^	0.1	221, 203, 178, 149, 135, 111, 95, 57	(+)	
75	13.02	Curcumenol	235.1685	C_15_H_23_O_2_^+^	2.3	217, 199, 189, 179, 177, 161, 149, 121, 119, 113, 107, 105, 97, 95, 93, 85, 79, 69, 65, 55	(+)	(+)
76	13.14	Curcumenone	235.16878	C_15_H_23_O_2_^+^	0.4	207, 189, 165, 151, 149, 123, 119, 107, 83, 71	(+)	
77	13.32	Zederone	247.1319	C_15_H_19_O_3_^+^	3	229, 201, 183, 139, 123, 121, 107, 95, 81, 67, 65	(+)	(+)
78	13.7	Curdione	237.1843	C_15_H_25_O_2_^+^	2.3	221, 219, 201, 191, 135, 121, 109, 107, 93, 81, 69, 55	(+)	(+)
79	13.76	Germacrone/Zerumbone	219.1739	C_15_H_23_O^+^	1.9	203, 159, 135, 121, 107, 97, 93, 79, 69	(+)	(+)
80	14	Aerugidiol	249.1493	C_15_H_21_O_3_^−^	3	231, 223, 213, 205, 189, 184, 177, 165, 147, 113, 87, 79.9, 68, 55	(+)	(+)
81	14.14	Epi/Curzerenone	231.1374	C_15_H_19_O_2_^+^	2.1	213, 203, 198, 189, 183, 173, 161, 149, 135, 119, 105, 95, 83	(+)	
**IX- Fatty acids/esters**								
82	6.61	Suberic acid	173.0806	C_8_H_13_O_4_^−^	0.8	129, 111, 83, 67, 57	(+)	(+)
83	7.99	Azelaic acid	187.0963	C_9_H_15_O_4_^−^	0.6	169, 143, 125		(+)
84	11.4	Trihydroxyoctadecenoic	329.2327	C_18_H_33_O_5_^−^	1.4	229, 211, 183, 171	(+)	
85	12.8	Trihydroxyoctadecanoic acid	331.2482	C_18_H_35_O_5_^−^	1	313, 295, 277, 185, 127		(+)
86	13.3	Gingerglycolipid A	675.3589	C_33_H_55_O_14_^−^	0.4	415, 397, 277, 255, 235, 161, 101	(+)	
87	13.34	Dihydroxyoctadecadienoic acid	311.2219	C_18_H_31_O_4_^−^	0.9	293, 275, 249, 183, 171	(+)	
88	13.4	Hydroxy-oxopentadecanoic acid	271.1908	C_15_H_27_O_4_^−^	1.7	253, 227, 209, 97	(+)	(+)
89	13.56	Hydroxy-oxopentadecadienoic acid	267.1594	C_15_H_23_O_4_^−^	1.4	249, 223, 205, 197, 181, 137, 97, 85	(+)	
90	13.79	Hydroxy-oxohexadecanoic acid	285.2064	C_16_H_29_O_4_^−^	1.3	267, 223, 59		(+)
91	14.1	Glyceropalmitoyl-*O*-dihexoside	653.3743	C_31_H_57_O_14_^−^	0.07	415, 397, 323, 255, 179, 89	(+)	
92	14.18	Palmitoyl-sn-glycero-phosphoethanolamine (LysoPE(0:0/16:0)	452.2775	C_21_H_43_NO_7_P^−^	0.8	339, 255, 196	(+)	
93	14.2	Oleoyl glycero-phosphoethanolamine	478.2931	C_23_H_45_NO_7_P^−^	0.7	339, 281, 196	(+)	
94	14.23	Hydroxy octadecadienoic acid	295.2271	C_18_H_31_O_3_^−^	1.2	277, 195	(+)	(+)
95	15	Hydroxypalmitic acid	271.2270	C_16_H_31_O_3_^−^	1	253, 225		(+)
96	17	Oleoyl-*O*-methyl-glycero-phosphate	449.2666	C_22_H_42_ O_7_P^−^	0.8	281, 167	(+)	
97	17.02	Dilinoleoyl-glycero-phosphate	695.4646	C_39_H_68_ O_8_P^−^	0.04	415, 279, 153	(+)	
98	17.04	Palmitoyl-linoleoyl-glycero-phosphoinositol	833.5173	C_43_H_78_ O_13_P^−^	0.15	553, 391, 279, 255	(+)	(+)
99	17.06	Dioleoylglycerophosphate	697.4793	C_39_H_69_ O_8_P^−^	0.53	415, 280	(+)	(+)
100	17.07	Linoleoyl-glycerophosphate	433.2352	C_21_H_38_ O_7_P^−^	0.6	279, 153	(+)	
101	17.1	Palmitoyl-linoleoyl-glycero-phosphate	671.4642	C_37_H_68_ O_8_P^−^	0.5	415, 391, 279, 255	(+)	(+)
102	17.2	Palmitoyl-oleoyl-glycerophosphoinositol	835.5312	C_43_H_80_O_13_P^−^	2	553, 281, 255	(+)	(+)
**X- Miscellaneous**								
103	1.1	Uridine	243.0614	C_9_H_11_O_6_N_2_^−^	0.9	183, 153, 140, 113, 110	(+)	
104	3.12	Methyl Uridine	257.0772	C_10_H_13_O_6_N_2_^−^	1.6	228, 214, 183, 155, 139, 124, 109	(+)	
105	3.59	Panthenol	204.1227	C_9_H_18_NO_4_^−^	1.6	125, 102, 71, 59	(+)	
106	3.71	Pyroglutamic acid	128.0339	C_5_H_6_NO_3_^−^	2	98, 81, 65	(+)	
107	4.1	Unknown	271.1181	C_13_H_19_O_6_^−^	1.7	193, 177, 135	(+)	(+)
108	4.9	Unknown	425.2019	C_18_H_33_O_11_^−^	0.5	293, 263, 209	(+)	
109	5.81	Hydroxy-trimethyl-cyclohexenoic acid-O-hexoside (picrocrocinic acid)	345.1547	C_16_H_25_O_8_^−^	1.03	179, 165, 161, 149, 137, 123, 101, 89		(+)
110	5.82	Dihydroxy-megastigmadien-one-*O*-hexoside (blumenol hexoside)	385.1866	C_19_H_29_O_8_^−^	2.4	223, 205, 161, 153, 101		(+)

Overall, UPLC/MS analysis revealed 110 metabolites including 7 sugars, 4 organic acids, 10 phenolics, 5 cinnamates, 5 flavonoids, 1 anthocyanin, 2 alkaloids, 23 di/aryl alkanoids, 24 sesquiterpenes, 21 fatty acids/glycerolipids, and 8 miscellaneous compounds (Table 2). Common fragmentation pathways in MS/MS spectra included dehydration (-H_2_O, 18 amu), deglycosilation (-C_6_H_10_O_5_, 162 amu for hexose, C_5_H_8_O_4_, 132 amu for pentose, -C_6_H_10_O_4_, 146 amu for deoxyhexose), decarboxylation (-COO, 44 amu), demethoxylation (-OCH_3_, 30 amu), decarbonylation (-CO, 28 amu), dealkylation, retro-Diels-Alder (RDA), and ring cleavage, as discussed in detail in the next subsections. UPLC/HR-MS/MS analysis provided insight into the chemical reservoir of *C. caesia* with several detected metabolites are reported herein for the first time, and adding to that taxa rich chemical composition.

#### Organic acids

Low molecular weight organic acids play a role in rhizomes to alleviate microbial stress and reduce heavy metals absorption^40^. Herein, 4 organic acids were detected in blue curcuma, viz. malic, citric, succinic and benzoic acids in peaks 1–4 early eluted at Rt range 0.9–1.7 min (Table 2)^4^. Observed fragments included decarboxylated and dehydrated product ions matching databases. For example, citric acid [M-H]^−^191.01881, C_6_H_7_O_7_^−^ showed dehydrated ion at *m/z* 173, C_6_H_5_O_6_^−^, followed by decarboxylation at *m/z* 129, C_5_H_5_O_4_^−,^ and further fragments at *m/z* 111, *m/z* 103, and *m/z* 87.

#### Phenolics, flavonoids, and anthocyanins

Phenolics alongside flavonoids are biosynthesized in plants as secondary metabolites mainly to filter or screen ultraviolet rays, alleviating its harmful oxidative stress, and as attractants for pollinators; thus, their abundance in rhizomes is limited relative to leaves^41^. In *Curcuma*, only 14 flavonoids were previously reported^2^. As shown in Tables 2 and 10 phenolics, 3 flavonoids, and 1 anthocyanin were annotated in *C. caesia* versus 3 phenolics and 3 flavonoids in *C. longa;* matching references reported on genus *Curcuma*^3,6,42^.

Interestingly, a *p*-hydroquinone derivative was detected exclusively in *C. caesia*, peak 16, at [M-H]^−^ 331.10275, C_14_H_19_O_9_^−^ for the first time (Table 2). Such compounds are potent ferric-ion reductants to its GIT-absorbable ferrous form, decolorizing agents, skin lightening agents, anti-inflammatory and potent free radical scavengers^43–47^. Fragmentation pathways yielded deglycosilated product ion at *m/z*168, C_8_H_8_O_4_^−^, followed by sequential demethylation at *m/z* 153, C_7_H_5_O_4_^−^ and *m/z* 138, C_6_H_2_O_4_^−^, further fragments at *m/z* 125, *m/z* 97, *m/z* 93, *m/z* 83, *m/z* 71 and *m/z* 59 corresponding to dehydroxylation and hydroquinone ring cleavage, matching the spectrum of leonuriside A, that is dimethoxy-hydroquinone-*O*-hexoside (Suppl.Fig.S5), as reported in literature and databases^48^.

Flavans and flavanones were major flavonoid subclasses detected in *C. caesia* in peaks 22, 25 and 26, whereas, *C. longa* had additional flavonols in peaks 22, 24 and 27 (Table 2)^2,42^. Pinocembrin flavanone in peak 27 was annotated herein in *C. longa* extract only at [M-H]^−^ 255.0654, C_15_H_11_O_4_^−^, known to exert several health benefits^49,50^.

In contrast, malvidin-*O*-rutinoside, detected in peak 23 exclusively in *C. caesia* for the first time, is an anthocyanin that imparts violet color and was previously reported in *C. alismatifolia* pink bracts^2^, and could contribute to the blue coloration of *C. caesia* alongside azulenes (Suppl.Fig.**S1**). The molecular ion was detected only in positive ionization mode at [M + H]^+^ 639.19128, C_29_H_35_O_16_^+^ (Suppl. Fig. S6), which yielded fragments at *m/z* 493, C_23_H_25_O_12_^+^ and *m/z* 331, C_17_H_15_O_7_^+^ for the sequential deglycosilation of deoxyhexose and hexose sugars, respectively, followed by RDA fragmentation of malvidin at *m/z* 153^51^ (Table 2).

#### Cinnamates

In this study, 5 cinnamic acid derivatives were detected in peaks 28–32 (Table 2). MS/MS Spectra in peaks 28, 31 and 32 showed feruloyl fragment ion at *m/z* 193, C_10_H_9_O_4_^−^, while peaks 29 and 30 had molecular ion at [M-H]^−^ 179.0335, C_9_H_7_O_4_^−^ and [M-H]^−^ 147.04379, C_9_H_7_O_2_^−^, identified as caffeic and cinnamic acids, respectively. Likewise, ferulic acid derivative in peak 31 annotated as feruloyloxy-methoxycinnamic acid at [M-H]^−^339.0862, C_19_H_15_O_6_^−^, was previously reported in *Curcuma* but first time in *C. longa* rhizome^6^. Another feruloyl derivative in peak 32 in *C. caesia* was annotated as oxyprenylated derivative of ferulic acid, namely, methylbutenoxy-ferulic acid, reported for the first time in *Curcuma* taxa. In detail, molecular ion was detected at [M-H]^−^ 261.1124, C_15_H_17_O_4_^−^ that yielded upon fragmentation several product ions viz. decarboxylated ion at *m/z* 217, C_14_H_17_O_2_^−^, feruloyl moiety at *m/z* 193, C_10_H_9_O_4_^−^ upon the loss of the prenyl side chain (C_5_H_8_), *m/z* 149 C_9_H_9_O_2_^−^ (-OCH_3_ & loss of oxyprenyl sidechain), *m/z* 121, C_8_H_9_O^−^ and *m/z* 105 C_8_H_9_^−^ (-CO_2_, -OCH_3_ &loss of pentenyl side chain) followed by dealkylation at *m/z* 79, C_6_H_7_^−^ whereas, the isopentenyl side chain was detected at *m/z* 81, C_5_H_5_O^−^ and its dealkylated fragment at *m/z* 67, C_4_H_3_O^−^ (Table 1, Suppl.Fig.S7).

#### Alkaloids

In previous studies, *C. longa* was reported to yield hydroxyquinoline alkaloids^2,6^. Herein, 2 hydroxyquinoline alkaloids are reported for the first time in both blue and yellow curcuma as shown in peaks 33 and 34 at [M-H]^−^ 366.0824, C_16_H_16_NO_9_^−^ and [M-H]^−^ 382.0771, C_16_H_16_NO_10_^−^annotated as di- and tri-hydroxyquinoline carboxylic acid-*O*-hexoside, respectively (Table 2). Fragments aiding in identification included deglycosilation (−162 amu, C_6_H_10_O_5_), decarboxylation (−44 amu, CO_2_) and quinoline ring cleavage matching spectra on databases^52^ (Suppl.Fig.S8). Similar structures were reported previously in *Ephedra* and cereals to which beneficial health promoting effect was attributed^53,54^.

#### *Di/Aryl alkanoids* (*Curcuminoids*,* bisabolane-sesquiterpene derivatives)*

Aryl and diaryl alkanoids are characteristic polyphenols in *Curcuma*, particularly in *C. longa*. The curcuminoids are diaryl-heptanoids, classified as bisabolane-type of sesquiterpenes. It imparts the distinguished yellow color and spice flavor of yellow curcuma and to which several therapeutic effects are attributed^3^. In this study, 23 di/aryl alkanoids were detected, predominantly in *C. longa* rhizome as shown in peaks 35–57 through Rt range 9.5–14.9 min, and were indeed previously reported in yellow curcuma^2,3,6,42,50^. In contrast, only 9 curcuminoids were detected in *C. caesia* as in peaks 35, 40, 45, 46, 47, 52, 53 and 54 (Table 2), and suggestive for activation of cucruminoids biosynthesis in *C. longa* as official drug in that taxa. Other diaryl alkanoids included furanone and pentadienone derivatives as in peaks 39, 41–43, 48 and 52 (Table 2).

Identification was based on reported fragmentation pathways and diagnostic product ions as explained in detail by Rasheed, et al., and Quirós-Fallas, et al.^3,50^, (Suppl. Fig. S9). For example, C_3_- and C_5_- di-keto curcuminoids such as curcumin I, II, III and their analogues, undergo heptanoid chain cleavage at C_4_, followed by decarbonylation (-CO, 28 amu), demethylation and dehydroxylation (-CH_3_-OH, 32 amu) to yield diagnostic ions, depending on aryl substitution, commonly detected at *m/z* 177, *m/z* 147, *m/z* 145, *m/z* 119^3,50^. In case of a single C_3_-keto alkanoids as in peaks 49 and 52, diagnostic product ion at *m/z* 137 for the neutral loss of substituted aryl moiety was observed as a result of precursor ion rearrangement followed by chain cleavage at C_1_-C_2_ bond^3,50^. In mono- or di-hydroxylated heptanoids, dehydrated product ion was detected (-H_2_O, 18 amu). Whereas, cucruminoids having furanone ring in the central chain as curcumalongin A/B/C and others, as in peaks 39, 41–43 and 48 (Table 2), fragmentation pathway included cleavage of the 5-membered furanone ring preceded by precursor ion rearrangement to yield product ion at *m/z* 153 or *m/z* 123 ^3^. Finally, the C_3_-ester containing curcuminoids as in calebin A, peak 37 (Table 2) had cleavage at the ester linkage releasing diagnostic ions at *m/z* 177 and *m/z* 207 ^3^. The mono-aryl heptanoids were detected exclusively in yellow curcuma at [M + H]^+^ 217.1579; C_15_H_21_O^+^ and 219.174; C_15_H_23_O^+^ in peaks 56 and 57, and were identified as turmerone and dihydroturmerone having diagnostic ions at *m/z* 119 and *m/z*121, respectively^50^, (Table 2).

#### Sesquiterpenes

Sesquiterpenes are the major phytochemicals detected in genus *Curcuma*, where 328 compounds belonging to various subclasses were reported among 28 species^2^. In this study, 24 sesquiterpenes were tentatively identified in blue curcuma; peaks 58–81, several of which are reported for the first time in *C. caesia.* In contrast, only 7 of them were detected in yellow curcuma, peaks 65, 66,75, 77–80 (Table 2). Herein, sesquiterpenes were 5 germacrane (peaks 72, 73, 77–79), 1 elemane (peak 81), 1 carabrane (peak 76) and 17 guainane (guainolide) skeletons (Table 2) aside from the bisabaolane-type that was discussed in the previous subsection 3.2.5^2,9,55^. *C. longa* had 2 types only i.e., 3 germacrane and 4 guainane. In contrast, *C. caesia* possessed the 4 subtypes of sesquiterpenes consistent with previously reported literature (Fig. 3)^2,9,55,56^.

MS/MS Spectra distinguished between sesquiterpenes *via* observing product ions and their respective elemental formulae, that correspond to dehydration, decarboxylation, dealkylation and, most importantly, RDA fragmentation that allowed to discriminate between the different subtypes. For example, peaks 72, 75 and 76 had the same molecular ion and formula at [M + H] ^+^ 235.1685, C_15_H_23_O_2_^+^; the first, peak 72, showed fragments for the loss of water, carbonyl and isopropenyl side chain at *m/z* 217; C_15_H_21_O^+^ (-H_2_O, 18 amu), *m/z* 207; C_14_H_23_O^+^ (-CO, 28 amu), *m/z* 189; C_14_H_21_^+^ (-H_2_O and -CO), *m/z* 193; C_12_H_17_O_2_^+^ (- isopropenyl C_3_H_6_^+^) and *m/z* 147; C_11_H_15_^+^ (loss of H_2_O, CO and isopropenyl) followed by sequential delalkylation at *m/z* 135, *m/z* 121, *m/z* 107, *m/z* 93 and *m/z* 79 which is consistent with germacrone epoxide (germacrane type) (Suppl. Fig. S10A). The second, peak 75, showed dehydrated ion at *m/z* 217; C_15_H_21_O^+^, pentyl-ring cleavage at *m/z* 179; C_11_ H_15_O_2_^+^, and *m/z* 55; C_4_H_7_^+^, RDA fragmentation of heptyl-ring at *m/z* 113; C_6_H_9_O^+^_2_, *m/z* 97; C_6_H_9_O^+^ and *m/z* 69; C_5_H_9_^+^, which is consistent with the guainane-type sesquiterpene curcumenol (Suppl. Fig. S10B). Whereas, the carabrane-type curcumenone in peak 76 showed product ions for decarbonylation at *m/z* 207; C_14_H_23_O^+^ (-CO, 28 amu), butanone-side chain cleavage at *m/z* 165; C_11_H_17_O^+^ and *m/z* 71; C_4_H_7_O^+^, propyl-ring cleavage and loss of butanone-side chain at *m/z* 151, C_10_H_15_O^+^ and *m/z* 85, loss of isopropenyl and butanone side chain at *m/z* 123; C_8_H_11_O^+^ (Suppl. Fig. S10C). In peak 81, epi/curzerenone is an elemane sesquiterpene that was previously reported in *C. caesia*^9^. The precursor ion was detected at [M + H]^+^ 231.1374; C_15_H_19_O_2_^+^, which upon fragmentation resulted in diagnostic product ions at *m/z* 123; C_7_H_7_O_2_^+^ and *m/z* 109; C_8_H_13_^+^ for the hexyl-ring cleavage, *m/z* 173; C_12_H_13_O^+^ and *m/z* 55; C_3_H_3_O^+^ for furanone ring cleavage, *m/z* 161 C_10_H_9_O_2_^+^ for the loss of isopropenyl and ethenyl substituents followed by further dealkylation at *m/z* 149 and *m/z* 137. Furthermore, product ion corresponding to the furanone ring was detected at *m/z* 83; C_5_H_7_O^+^, and the hexanone ring moiety at *m/z* 95; C_6_H_7_O^+^.

Zedoalactones and zedoarolides are guaianolides that were previously reported in *Curcuma*, including *C. longa*^50^. Whereas, the dihydro-derivative of zedoarolide B, peak 69, detected in *C. caesia* is, according to our knowledge, a newly detected metabolite (Table 2). The precursor ion was detected in negative ionization mode at [M-H]^−^ 283.1543; C_15_H_23_O_5_^−^, having extra 2-hydogen atoms than zedoarolide B, peak 66, that was previously reported in *C. phaeocaulis and C. zedoaria*^2,6,55^. Fragmentation yielded product ions corresponding to decarboxylation (-CO_2_, 44 amu) and 3 successive dehydration (−3 × 18 amu) at *m/z* 265; C_15_H_21_O_4_^−^, *m/z* 239; C_14_H_23_O_3_^−^, *m/z* 221; C_14_H_21_O_2_^−^, *m/z* 203; C_14_H_19_O^−^ and *m/z* 185; C_14_H_17_^−^ (Suppl. Fig.S11). Other fragments included lactone ring cleavage at *m/z* 193, C_12_H_17_O_2_^−^, followed by sequential dealkylation and dehydration at *m/z* 169, C_10_H_17_O_2_^−^, *m/z* 151; C_10_H_15_O^−^ and *m/z* 133, C_10_H_13_^−^.

The myriad chemical reservoir of sesquiterpenes in *C. caesia* is a special feature that definitely has an impact on its therapeutic effects, particularly, anticancer, anti-inflammatory, antimicrobial and hepatoprotective bioactivities among several others that are reported on each of its subclasses *via* different mechanisms of action^55^. Further studies are recommended to compare the bioactivity of the crude extract containing mixture of sesquiterpenes with that of the isolated components or subfractions, as well as conducting chronic and acute toxicity studies to determine its recommended daily intake dose.

#### Fatty acids (FA)/Glycerolipids (GL)

Fatty constituents were one of the major classes detected herein represented by 21 metabolites in negative ionization mode as in peaks 82–103 and were eluted at wide Rt range 6.6–17.2 min according to their relative polarity i.e. short-chain dicarboxylated/oxygenated FA were eluted earlier in contrast to long-chain FA glycerates (Table 2). Several metabolites were derivatives of palmitic, linoleic and oleic acids as evidenced by product ions at *m/z* 255; C_16_H_31_O_2_^−^, *m/z* 279; C_18_H_31_O_2_^−^ and *m/z* 281; C_18_H_33_O_2_^−^, respectively. Fragmentation included dehydration (-H_2_O, 18 amu), decarboxylation (-COO, 44 amu) in addition to deglycosilation in case of glycated FA as in peak 91 detected in blue curcuma (Table 2). In phosphethanolamine derivatives, diagnostic ion at *m/z* 196 was detected as in peaks 92 and 93 (Table 2). Hydroxylated-oxo-penta/hexa-decanoic acids were detected in both species in peaks 88–90, in which hydroxy-oxohexadecanoic acid, peak 90, was previously reported in yellow curcuma^50^.

### Primary metabolites profiling of *C. longa* and *C. caesie via* GC-MS (Post-Silylation)

**Fig. 4:**
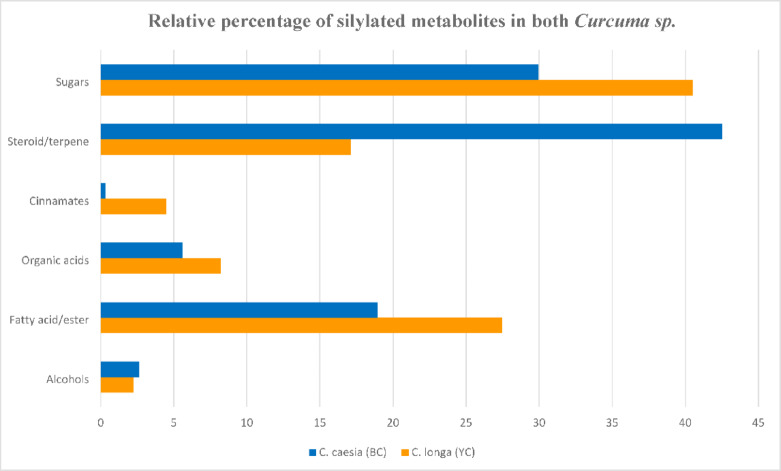
Relative percentage of silylated metabolites detected in *C. caesia* (blue) and *C. longa* (yellow) rhizomes *via* GC/MS analysis.

To assess variation of primary metabolites among *C. longa* and *C. caesie* to account for nutritive value and not readily ionized using ESI in UPLC-MS, GC–MS analysis was employed post-silylation (Suppl. Fig. S12). A total of 38 peaks were identified (Table 3) categorized into alcohols (2), fatty acids/esters (8), organic acids (5), sugars (7), alongside cinnamates (3), steroids/terpenes (13) as secondary metabolites. *C. longa* exhibited higher sugar level (40.5%), followed by fatty acids/esters (27.4%). In contrast, *C. caesia* was dominated by steroids/terpenes (42.5%), with sugars (29.9%) as the second major component (Fig. 4), and to rationalize why *C. longa* is more suited for culinary uses than *C. caesia*. Differences among each class is explained below in more details in the next subsections.

**Table 3:** Relative percentiles of silylated compounds detected in *C. caesia* and *C. longa via* GC/MS analysis.

Peak	Rt (min.)	KI	Identification	C. longa	C. caesia
**Sugars**					
1	14.9	1814.5	D-Fructose, 5TMS *	0.02 ± 0.003	0.05 ± 0.03
2	15.3	1854	Methyl.alpha.-Arabinofuranoside, 3TMS	0.09 ± 0.05	0.32 ± 0.04
3	15.90	1904.7	Glucose, 5TMS *	5.59 ± 0.44	5.86 ± 1.09
4	16.1	1915	Methyl pentopyranoside	2.27 ± 0.23	1.21 ± 0.33
5	16.7	1989.8	b-Glucopyranose, 5TMS	0.53 ± 0.05	0.35 ± 0.26
6	16.7	1989	Talose, 5TMS	11.97 ± 1.15	10.98 ± 2.35
7	22.4	2674	Sucrose, 8TMS *	20.02 ± 1.15	11.16 ± 1.56
**Total sugars**				**40.5**	**29.93**
**Organic acids**					
8	13.6	1690	2-Methyl-2-butenedioic acid dimethyl ester	2.63 ± 0.2	1.78 ± 1.22
9	5.15	1063.1	Lactic Acid, 2TMS*	1.72 ± 0.71	0.84 ± 0.27
10	7.87	1237.4	Benzoic Acid, TMS	0.14 ± 0.01	0.57 ± 0.54
11	11.2	1487.5	Malic acid, 3TMS	1.37 ± 0.04	0.86 ± 0.21
12	13.09	1641	Glutaric acid, di(isobutyl) ester	2.35 ± 0.13	1.56 ± 1.06
**Total organic acids**				**8.2**	**5.61**
**Alcohols**					
13	8.3	1272.1	Glycerol, 3TMS *	1.79 ± 0.12	2.36 ± 0.67
14	12.0	1553	1-Dodecanol, TMS	0.45 ± 0.04	0.28 ± 0.24
**Total Alcohols**				**2.24**	**2.64**
**Cinnamates**					
15	14.3	1751	Hydrocinnamic acid 2TMS	0.09 ± 0.005	0.11 ± 0.03
16	16.1	1929	4-Coumaric acid, 2TMS	0.16 ± 0.03	0.15 ± 0.09
17	17.6	2082	(Iso)ferulic acid, 2TMS	4.22 ± 0.42	0.06 ± 0.03
**Total cinnamates**				**4.47**	**0.32**
**Steroid/terpene**					
18	11.3	1493.7	Curzerene	0.037 ± 0.01	1.55 ± 0.9
19	12.6	1599.3	Epicurzerenone	0.09 ± 0.02	22.29 ± 4.44
20	13.2	1657	aR-Turmerone	7.13 ± 1.9	0.14 ± 0.05
21	13.6	1695	Curlone	3.59 ± 0.84	0.21 ± 0.04
22	13.7	1698.1	Germacrone	3.59 ± 0.84	0.21 ± 0.04
23	13.9	1718	Neocurdione	0	0.01 ± 0.01
24	14.1	1739	b-Eudesmol, TMS	0.17 ± 0.05	1.02 ± 0.3
25	14.3	1718.4	Curdione	0.48 ± 0.04	0.68 ± 0.15
26	15.18	1832.8	Curcumenone	1.25 ± 0.15	0.19 ± 0.03
27	15.3	1845	4,5-Epoxygermacrone	0.16 ± 0.01	12.14 ± 2.49
28	16.8	1994.3	Zederone	0.11 ± 0.07	1.89 ± 0.33
29	17.7	2101.8	Valerenic acid, TMS	0.43 ± 0.14	2.16 ± 0.74
30	28.2	3467	b-Sitosterol, TMS	0.06 ± 0.02	0.04 ± 0.02
**Total steroid/terpene**				**17.1**	**42.54**
**Fatty acids/esters**					
31	17.08	2025	Palmitic Acid, TMS *	14.21 ± 1.16	10.24 ± 6.1
32	18.6	2190	Linoleic acid, TMS	1.11 ± 0.04	0.82 ± 0.07
33	18.6	2201.8	Oleic Acid, (Z)-, TMS	0.15 ± 0.01	0.1 ± 0.04
34	18.71	2201.9	11-Octadecenoic acid, (Z)-, TMS	1.11 ± 0.11	0.77 ± 0.5
35	18.8	2219	Stearic acid, TMS	6.4 ± 0.59	4.68 ± 3.05
36	21.7	2571	1-Monopalmitin, 2TMS	2.42 ± 0.87	1.29 ± 0.53
37	22.9	2571	1-Monolinolein, 2TMS	0.18 ± 0.01	0.12 ± 0.04
38	23.13	2763	Glycerol monostearate, 2TMS	1.92 ± 1.27	0.93 ± 0.64
**Total fatty acid/ester**				**27.49**	**18.95**

*denotes compounds confirmed using standards.

#### Sugars

Sugars amounted as the main primary metabolite class in *C. longa* and *C. caesie* represented by a total of 7 peaks belonging to mono- and di-sugars. The relative higher sugar level in *C. longa* at 40.5% versus 29.93% in *C. caesia* infers for the improved taste and nutritive value *in C. longa*. Among sugars, sucrose was the most abundant sugar detected at 20.02% and 11.16% in *C. longa* and *C. caesia*, respectively. The presence of sucrose at substantial levels suggest for its role as sweat attribute in both species^29,57^. Following sucrose, talose was another major sugar detected at 10–12%, less common in dietary sources, though of potential health benefits. Compared with di-sugars that showed differences among species, mono-sugar represented by glucose was present at comparable levels ca. 5–6%. These results fall in accordance with previous proximate analysis of *C. caesia* and *C. longa* revealing substantial sugar content (19–21%) of dry weight^58^, comparable to levels detected using GC/MS and extend here in to reveal for sucrose as the major form.

#### *Organic acids and alcohols*

Organic acids were detected in both species at 8.2% and 5.6% in in *C. longa* and *C. caesia*, respectively (Table 3). Organic acids contribute to food attributes as natural preservative, food digestion, and in protein utilization^59^, in addition to taste perception as a flavor enhancer. Major forms included 2-methyl-2-butenedioic acid dimethyl ester and glutaric acid, suggesting their potential use as food additive, preservation, and health-related effects. Compared to acids, alcohols, represented by glycerol and dodecanol, were detected at much lower levels of 2.2–2.6% (Table 3; Fig. 4). Previous studies reported the presence of various alcohols including glycerol and monoterpene alcohols, in *Curcuma* essential oils^60^.

#### Cinnamates

GC–MS analysis of *C. longa* and *C. caesia* extracts revealed much higher levels of cinnamates detected in C. *longa* than *C. caesia* at 4.5% and 0.3%, respectively. Examples of cinnamates included hydrocinnamic acid, 4-coumaric acid, and isoferulic/ferulic acid, with isoferulic/ferulic acid as major form in *C. longa* at 4.2%. The higher cinnamate level in *C. longa* suggests for stronger antioxidant and anti-inflammatory properties, as cinnamates are known for their role in human health^61,62^.

#### Steroids/terpenes

Steroids/terpenes were represented in *C. long* and *C. caesia* by 13 peaks, detected at much higher levels in *C. caesia* at 42.5% versus 17.1% in *C. longa*. Most notably, epicurzerenone was detected in *C. caesia* at 22.39% relative to *C. longa* at trace levels 0.09%. Epicurzerenone, a sesquiterpene, has been reported to exert potential antitumor and anti-inflammatory effects^63^. A study on essential oil analysis of *C. caesia* rhizomes reported less epicurzerenone (12.47%) comparable to levels detected here in this study^64^. Following epicurzerenone, 4,5-epoxygermacrone (12.14%) was the predominant sesquiterpene in *C. caesia*, suggesting a rich terpenoid profile associated with strong anti-inflammatory and antimicrobial activities^65^. Compared to *C. caesia*,* C. longa* is characterized by higher levels of αR-turmerone (7.13%) and curlone (3.59%), suggestive of differential terpenoids biosynthesis.

Other sesquiterpenes detected at much lower levels included 4,5-epoxygermacrone (12.1%), valerenic acid (2%), zederone (1.9%), and curzerene (1.6%) in *C. caesia*, as well as curlone (3.6%), germacrone (3.6%), and curcumenone (1.3%) in *C. longa*.

#### Fatty acids/esters

Fatty acids and esters represented the second and third most abundant metabolite class in *C. longa* and in *C. caesia*, respectively *(*Table 3). The higher fatty acid level in *C. longa* at 27.5% versus 19% in *C. caesia* highlights for improved nutritive value *in C. longa*, and is concurrent with the higher sugar profile. Among fatty acids/esters, palmitic acid was the major form detected at 14.2% and 10.2% in *C. longa* and *C. caesia*, respectively. The presence of palmitic acid at considerable levels in both *C. longa* and *C. caesia* suggests its role in energy metabolism^66^. A study on fatty acids composition of *C. longa* oil reported oleic acid likewise as the major component (56–58%)^67^, slightly higher than that detected herein in alcohol extract using GC/MS.Following palmitic acid, stearic acid was the other major fatty acid at 4–6%. Linoleic and oleic unsaturated fatty acids were detected at much lower levels in both species at 0.8–1.1%, and suggestive that saturated fatty acids predominate in *Curcuma* species. Asides from free fatty acids, acyl esters were likewise detected such as 1-monopalmitin, 1-monolinolein, and glycerol monostearate in both species at 0.1–2.1%.

### Aroma profiling of *C. longa* and *C. caesia* rhizomes *via* SPME coupled to GC-MS

**Fig. 5:**
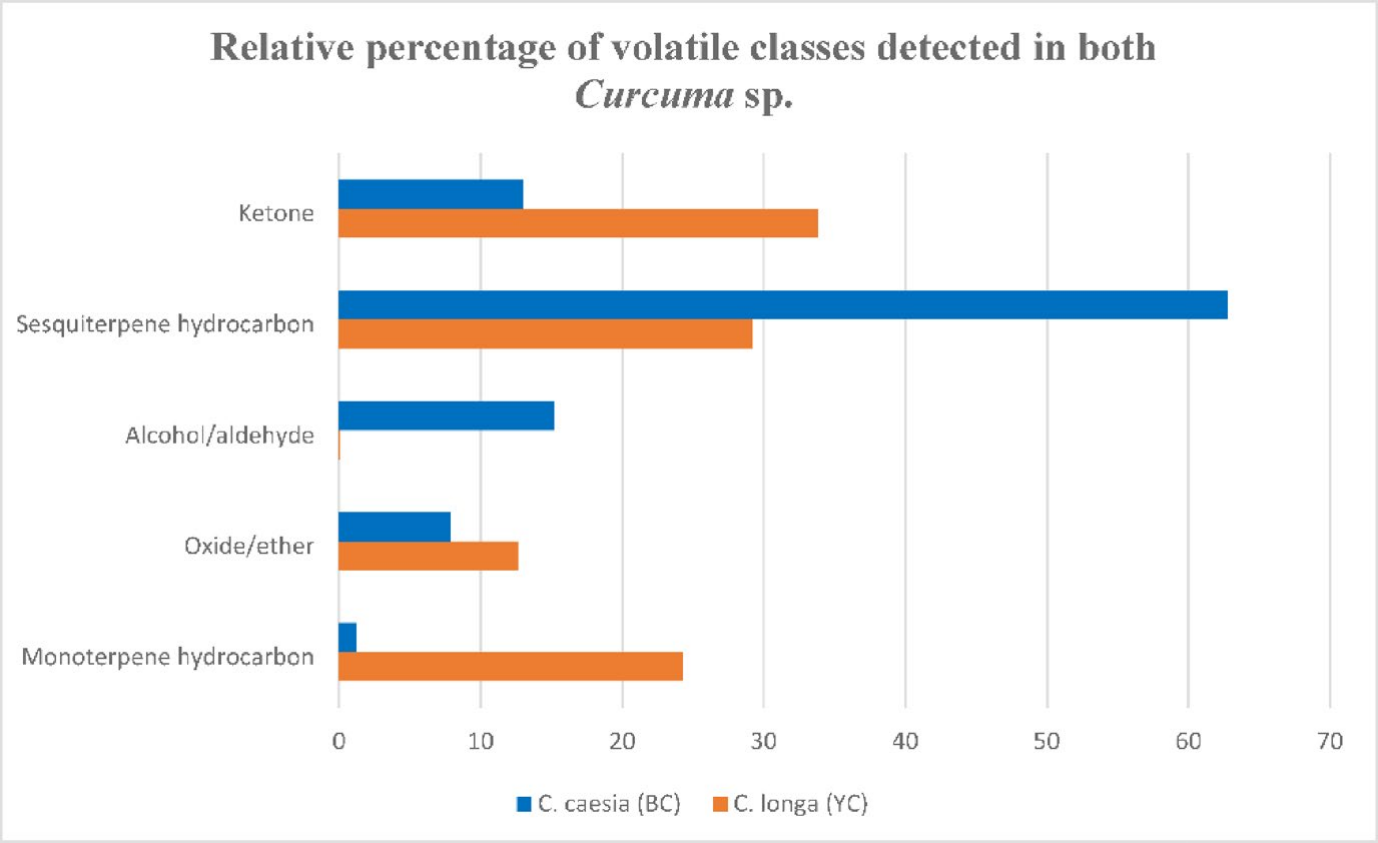
Relative percentage of aroma compounds detected in *C. caesia* (blue) and *C. longa* (yellow) *via* SPME-GC/MS analysis.

SPME is well suited for the profiling of aroma and was employed for *C. longa* and *C. caesia* rhizomes, providing the real composition of their volatile blends, and is first time to be reported in both species via SPME-GC/MS (Suppl. Fig. S13). A total of 26 volatile constituents were detected in both *C. longa* an*d C. caesia* rhizomes belonging to monoterpene hydrocarbon (6), oxide/ether (1), alcohol/aldehyde (2), sesquiterpene hydrocarbon (12), ketone (5), S-containing compounds (Suppl.Table 2). Both species showed comparable aroma composition, as seen in the relative percentile of each class in Fig. 5. In *C. longa*, ketones, and sesquiterpenes were the major volatile classes, detected at 33.8% and 29.17%, respectively, whereas sesquiterpenes were the most abundant in *C. caesia* (62.79%). Alcohol/aldehyde was detected in *C. longa* rhizomes at low levels (0.05%) versus monoterpenes detection at trace levels in *C. caesia* (1.18%). Tumerone was the major volatile in *C. longa* at 33.47% followed by β-sesquiphellandrene at 20.82%, while β-elemene was the major form in C. caesia at 18.64% followed by heptanol and zingiberene at %15.1 and 12.8%, respectively. Interestingly, turmerone ketones were reported to be responsible for *C. longa* aroma^68^, was detected at trace levels in *C. caesia* (0.01%). Turmerone was reported as a major constituent in the essential oil of *C. longa* at range 25.3–40.4%^69^, in accordance with our results using SPME. Likewise.

### Multivariate data analyses of blue curcuma *C. caesia* and yellow curcuma *C. longa* derived metabolome profiles

Classification models were attempted from the different analytical platforms i.e., UPLC/HR-MS/MS and GC-MS to identify discriminative metabolites for each species. Loading plots and S-plots identified biomarkers mediating for samples’ classification as denoted by their Mol.wt./Rt derived from respective PCA and OPLS-DA plots.

#### PCA and OPLS-DA analyses of UPLC/HR-MS/MS dataset

**Fig. 6:**
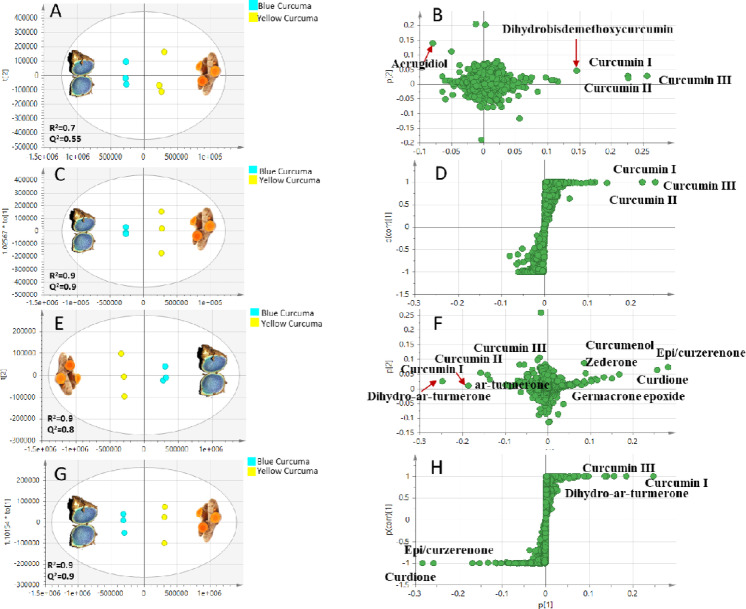
Multivariate data analyses of UPLC/HR-MS/MS dataset of blue curcuma (*C. caesia*) and yellow curcuma (*C. longa*) in both negative (**A-D**) and positive (**E-H**) ionization modes (*n* = 3). Principle component analysis (PCA) score plots (A, E) and loading plots (**B**,** F**); orthogonal partial least squares discriminant analysis (OPLS-DA) score plots (**C**,** G**) and S-loading plots (**D**,** H**) with PC1 and PC2 represent > 60% and > 90% of total variation in data derived from negative and positive ionization modes, respectively. Metabolites were assigned based on Mol.wt/Rt as identified in Table 1.

The unsupervised PCA model of UPLC/HR-MS/MS analyzed in negative ionization mode showed moderate prediction power (R2 = 0.7, Q2 = 0.55). Samples segregation was observed mostly along PC1 accounting for the major variance (Fig. 6A). PCA loading plot (Fig. 6B) revealed guainane-sesquiterpene “aerugidiol” as biomarker for *C. caesia* alongside other alkylbenzene sulfonate compounds at *m/z* 311 and *m/z* 325 (base peak *m/z* 183, C_8_H_7_O_3_S^−^). However, these are reported contaminants in irrigating waste water and pesticides used in agricultural field practice in China^70^. In contrast, *C. longa* was discriminated by exclusive presence of diarylheptanoids curcumin III and dihydrobisdemethoxycurcumin as well as abundance of curcumin I and curcumin II which was further confirmed using supervised OPLS-DA score plot (R2 = 0.9, Q2 = 0.9) and respective S-loading plot (Fig. 6C and D).

On the other side, the PCA model derived from UPLC/HR-MS/MS data analyzed in positive ionization mode showed stronger classification as evidenced by larger variance and prediction power (R2 = 0.9, Q2 = 0.9), where PC1 and PC2 accounted for ca. 90% of the variance (Fig. 6E). *C. longa* specimen was still discriminated by diaryl heptanoids curcumin I, curcumin II, and curcumin III (Fig. 6F**)** and in agreement with negative mode result **(**Fig. 6B**)**, in addition to monoarylheptanoids ar-turmerone and dihydro-ar-turmerone revealed in positive mode, likely due to their improved ionization in that mode and highlighting the benefit of acquisition in both modes. In contrast, *C. caesia* was distinguished by guainane, germacrane, and elemane types of sesquiterpenes, namely curcumenol, germacrone epoxide, zederone, curdione, and epi/curzerenone. Supervised OPLS-DA was further employed for its superiority in biomarkers assignment (R2 = 0.9, Q2 = 0.9) **(**Fig. 6G). The S-loading plot of metabolites verified curcumin I, curcumin III, and dihydro-ar-turmerone as biomarkers of *C. longa*, while curdione and epi/curzerenone were the discriminatory metabolites of *C. caesia* (Fig. 6H). The results are consistent with previous studies reporting that *C. caesia* is among 4 *Curcuma* species only that biosynthesize mixture of 4 different types of sesquiterpenes (guainane, germacrene, elemane and carabrane), whereas diaryl heptanoids are detected in 10 *Curcuma* species including *C. longa*^9^. These results confirm that chemotaxonomic classification in *Curcuma* taxa is based on diarylheptanoids versus sesquiterpenes biosynthesis.

#### PCA and OPLS-DA analyses of GC/MS silylated metabolite dataset

**Fig. 7:**
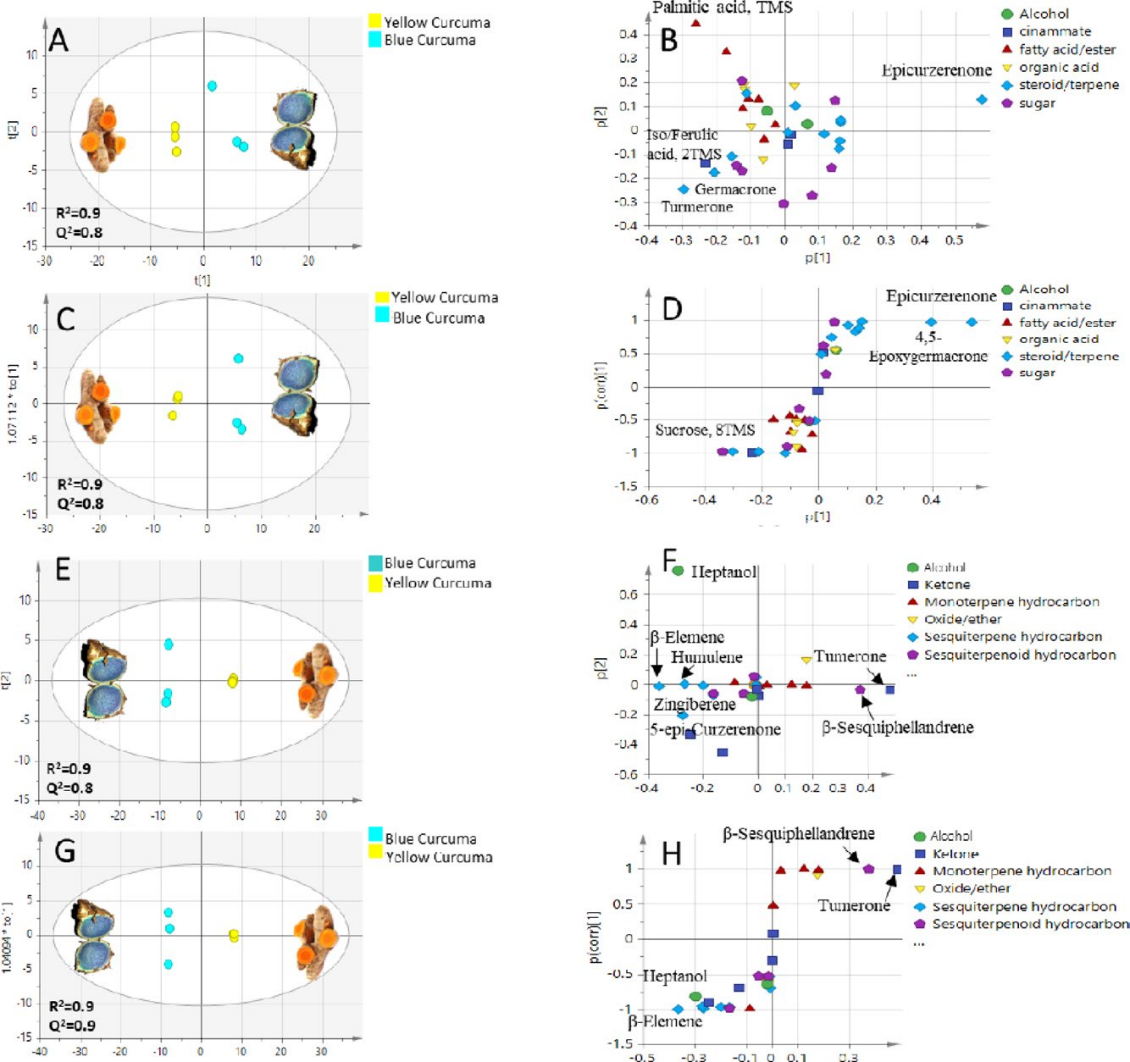
Multivariate data analyses of GC/MS dataset of blue curcuma (*C. caesia*) and yellow curcuma (*C. longa*) of silylated (**A-D**) and volatile (**E-H**) components (*n* = 3). Principle component analysis (PCA) score plots (**A**,** E**) and loading plots (**B**,** F**), orthogonal partial least squares discriminant analysis (OPLS-DA) score plots (**C**,** G**) and S-loading plots (**D**,** H**) with PC1 and PC2 represent > 70% and > 90% of total variation in data of silylated and volatile components, respectively. Metabolites were assigned based on Mol.wt/Rt as identified in Tables 2 and 3.

PCA model of silylated compounds to encompass a total of 39 peaks analyzed *via* GC/MS showed good variance and prediction power (R2 = 0.9, Q2 = 0.8) where PC1 and PC2 accounted for > 70% of metabolites (Fig. 7A). Loading plot of metabolites assigned epicurzerenone sesquiterpene as biomarker for blue curcuma *C. caesia*, while yellow curcuma *C. longa* was discriminated by abundance of palmitic acid, iso/ferulic acid, germacrone and arylheptanoid turmerone (Fig. 7B). These results are in agreement with UPLC/HR-MS/MS results shown in Table 1**and** Fig. 6E and H. To verify classification, OPLS-DA was conducted, where S-plot assigned epicurzerenone as *C. caesia* discriminatory metabolite alongside epoxygermacrone. In contrast, sucrose was abundant in *C. longa* (Fig. 7C and D), and to account for the improved taste of *C. longa* and culinary use compared to *C. caesia*.

#### PCA and OPLS-DA analyses of SPME-GC/MS volatiles dataset

With regards to volatile components analyzed *via* SPME-GC/MS, PCA and OPLS-DA score plots showed likewise good variance and prediction power (R2 = 0.9, Q2 = 0.8–0.9) representing > 90% of the total variance (Fig. 7E**).** Both loading and S-plots verified that turmerone and sesquiphellandrene are the discriminating volatiles of *C. longa* (Fig. 7F and H). PCA loading plot detected further enrichment of *C. caesia* aroma profile with alcohol i.e., heptanol, sesquiterpene hydrocarbons *β*-elemene, humulene, zingiberene and oxygenated ketone sesquiterpene epi/curzerenone (Fig. 7F). The S-plot verified further enrichment of *C. caesia* in heptanol and epi/curzerenone and considered its volatile biomarkers (Fig. 7H). It’s noteworthy that epi/curzerenone imparts a mushroom-like rooty odor of blue curcuma that is balanced with sweet and fresh herbal aroma attributed to sesquiterpenes hydrocarbons^71^. In contrast, turmerone essentially imparts the distinctive turmeric spicy odor in *C. longa*^72^.

In conclusion, epi/curzerenone oxygenated sesquiterpene of elemane type were recognized as a discriminating metabolite for blue curcuma *C. caesia* based on UPLC/HR-MS/MS, post- silylated GC/MS, and SPME-GC/MS analyses. In contrast, *C. longa* is recognized with its mono-aryl and diaryl-heptanoids, in addition to its further enrichment in fatty acids, sugars, and cinnamates (Figs. 6 and 7**).**

### Quantification of total phenolic (TPC) and flavonoid contents (TFC) in *C. caesia* and *C. longa* rhizomes

The total phenolic and flavonoid contents were quantified in both turmeric samples for standardization purposes. The results revealed significant differences in both contents, where TPC in yellow curcuma (106.26 ± 3.53 mg GAE/g w) was ~ 7 times higher than that in blue curcuma (16.64 ± 0.14 mg GAE/g w) quantified as gallic acid equivalent. Similarly, TFC in yellow curcuma (18.45 ± 0.3 mg RE/g w) exhibited triple the flavonoid content of blue curcuma (6.43 ± 0.27 mg RE/g w) quantified as rutin equivalent. The high TPC in yellow curcuma is compatible with its further enrichment with curcuminoids (Di/phenyl heptanoids) as revealed from UPLC/HR-MS/MS profiling and subsequent multivariate data analyses in contrast to blue curcuma (Table 2; Fig. 6).

### Bioactivity assays

#### Antioxidant activity determination

Scavenging of free radicals is known to mediate for several bioactivities and serve as a good prediction for exploring the potentials of natural resources, thus both species were comparatively assayed for their free radical scavenging potential using 3 different in vitro assays viz. DPPH, ABTS and FRAP, measured as trolox equivalent. This ensured to have better validated evaluation of both turmeric extracts according to their lipophilic and hydrophilic constituents using different action mechanisms^73^. Yellow curcuma exhibited higher scavenging activity by DPPH (37.36 ± 0.99 mg TE/g w) and ABTS (34.37 ± 0.74 mg TE/g w) assays than the blue curcuma (20.78 ± 0.74 mg TE/g w and 18.31 ± 0.18 mg TE/g w, respectively) which still showed moderate antioxidant activity. In contrast, FRAP assay which measures the reducing power or electron-donating capacity of compounds, revealed comparable antioxidant potential of blue (34.12 ± 0.33 mg TE/g w) and yellow curcuma (31.44 ± 0.93 mg TE/g w). The obtained results are compatible with the enriched phenolic content in yellow curcuma that are potent scavengers of free radicals, as well as, the presence of hydroquinone derivatives detected *via* UPLC/HR-MS/MS in blue curcuma that are potent ferric reductants, thus reflected in the FRAP assay^45^ (Table 2).

#### Antidiabetic activity determination via α-glucosidase Inhibition

Both blue and yellow curcuma are reported to exhibit in vivo antidiabetic activity^74,75^. In this study their α-glucosidase inhibition activity was determined comparably against acarbose reference drug. *C. longa* showed more potent activity evidenced by lower IC_50_ (0.325 ± 0.025 mg/ml) than that observed for *C. caesia* rhizome (1.04 ± 0.064 mg/mL) and acarbose (0.4891 ± 0.1 mg/mL). This is justified by the further abundance of phenolics in *C. longa* which interfere with the active sites of α-glucosidase, thus, preventing it from binding with carbohydrates^76^. Moreover, curcuminoids are proven by clinical and preclinical trials to act synergistically against diabetes *via* different mechanism of action^6,77^. On the other side, terpenes of *C. caesia* showed high in silico binding affinity to α-glucosidase enzyme that contributes in the herein observed antidiabetic activity^78^.

#### Anti-obesity activity determination via lipase Inhibition

Rhizome extracts of *C. longa* and *C. caesia* were investigated for their anti-obesity activity using orlistat reference drug *via* lipase inhibition. Both samples showed no significant inhibitory activity against lipase at the maximal test concentration (10 mg/mL) and its dilutions (orlistat IC_50_ = 0.16 ± 0.08 mg/mL).

## Conclusion

The study presented the first integrated comparative metabolome profiling of *C. caesia* and *C. longa* using multiplex approach aided by multivariate data analyses, uncovering distinct metabolic divergence among both species and highlighting several first-time reported metabolites in the less explored turmeric blue curcuma. The analyses revealed for 11 metabolites annotated *via* NMR, 110 tentatively identified metabolites by UPLC/HR-MS/MS, 38 silylated compounds and 26 volatiles detected by GC/MS analysis. Samples’ classification and discriminatory chemotaxonomic biomarkers were assigned for each species by PCA and OPLS data analyses emphasizing diverse sesquiterpenes in *C. caesia vs.* curcuminoids and sugar enrichment in *C. longa* aligning with its established culinary and therapeutic uses. Antioxidant and antidiabetic bioassays showed superior activity of *C. longa* compatible with its higher quantified contents of total phenolics and flavonoids, whereas both species were inactive as anti-obesity agents compared to orlistat reference drug. Targeted isolation of novel sesquiterpenes annotated in *C. caesia* is recommended for therapeutic evaluation to elucidate its potential in pharmaceutical and food industries.

## Supplementary Information

Below is the link to the electronic supplementary material.

Supplementary Material 1

## Data Availability

The raw files dataset used and/or analyzed during the current study available from the corresponding author on reasonable request.
