# Supplementary material for: NMR- and MS-based multiplex approach for comparative metabolome profiling of Curcuma caesia versus curcuma longa rhizomes and in relation to in vitro biological effects
Source: Sci Rep. 2025 Dec 18;15:44052. doi: 10.1038/s41598-025-30956-9 (PMC12715240; doi:10.1038/s41598-025-30956-9)
Supplement: Supplementary file 1 — Supplementary Material 1 [file 41598_2025_30956_MOESM1_ESM.docx]

**Supplementary file**

**NMR- and MS-based Multiplex approach for comparative metabolome profiling of *Curcuma caesia* *versus* *Curcuma* *longa* rhizomes and in relation to *in vitro* biological effects**

**
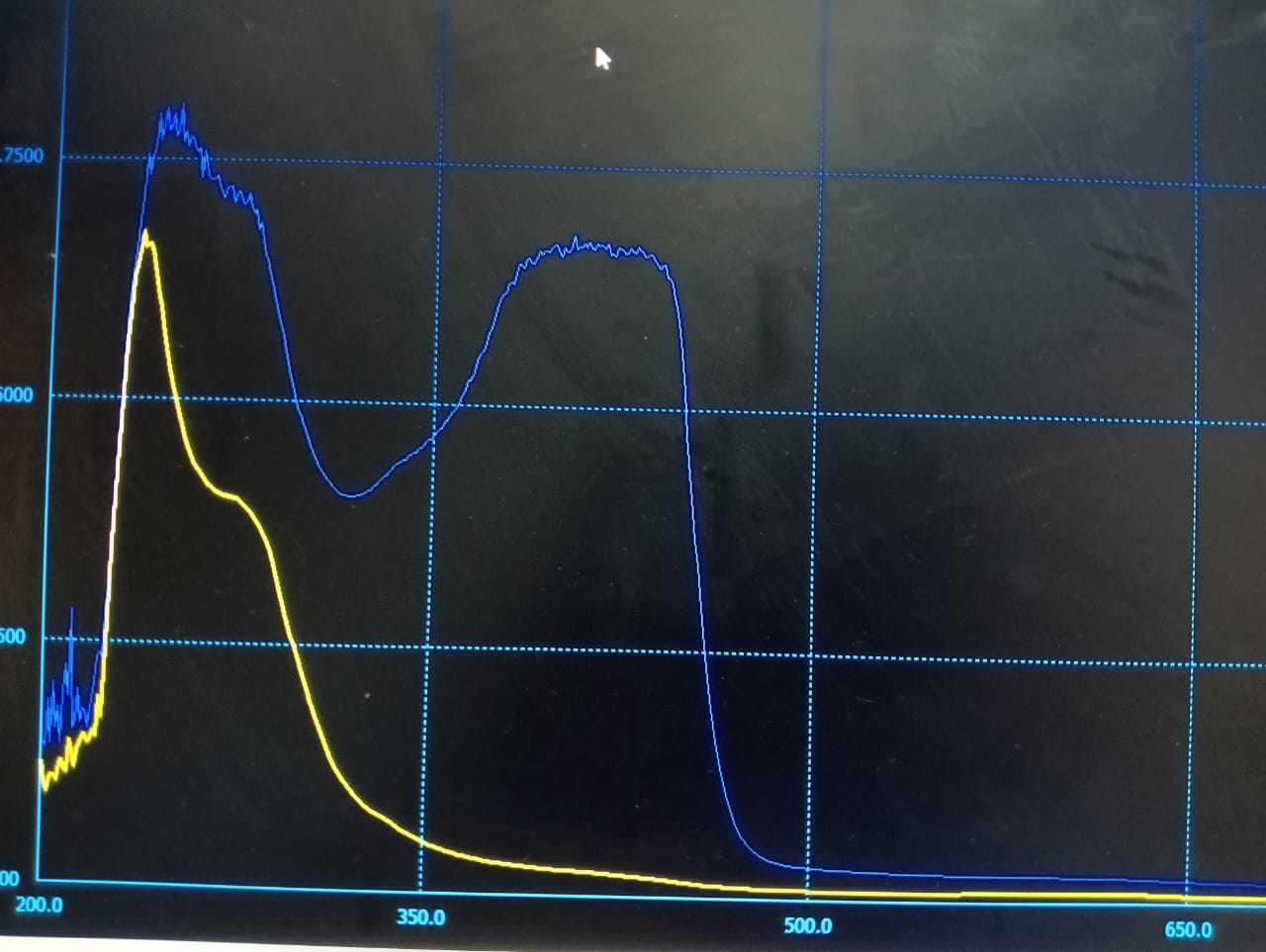
**

**400 nm**

**Suppl.Fig. S1**: UV absorbance of *C. caesia* (blue) and *C. longa* (yellow) chloroform extracts showing exclusive strong absorbance for blue curcuma at longer wavelength ca. λ_max_ 400 nm, that inferred the presence of guaiazulenes (alkylated derivatives of azulenes).


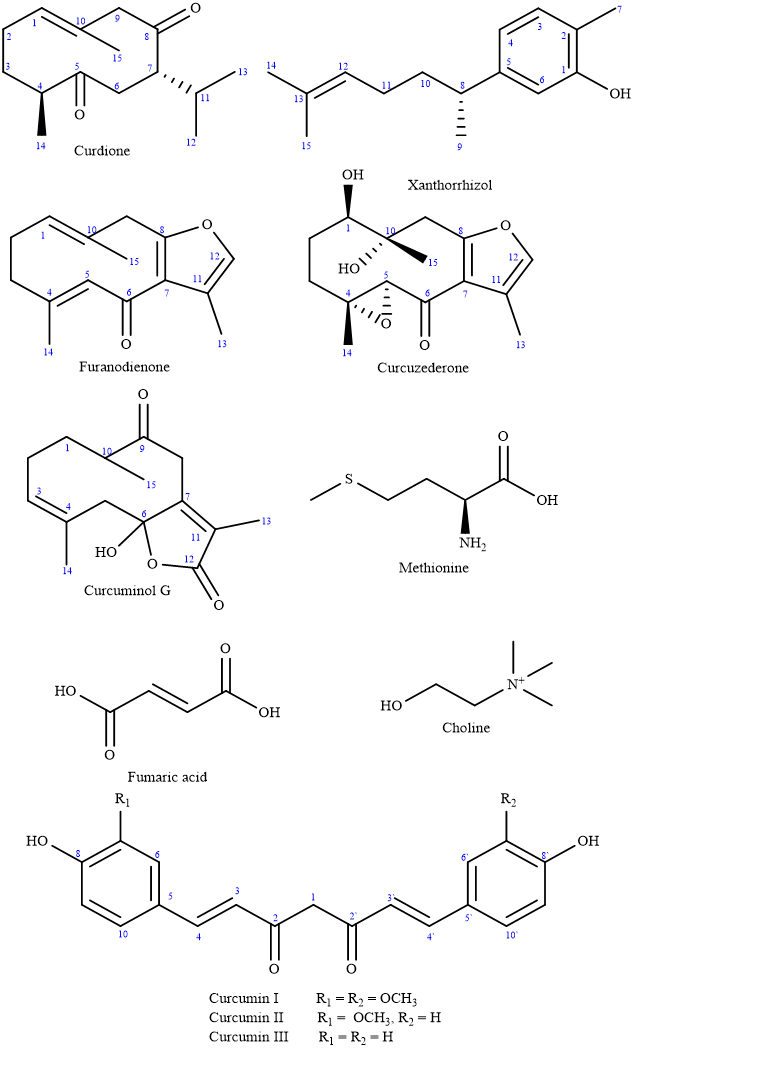


**Suppl.Fig. S2**: Structures of the major primary and secondary metabolites detected in methanol extracts of Curcurma caesia (BT), and *Curcuma longa* (YT) *via* 1D- and 2D-NMR spectroscopy.

**Suppl.Fig. S3: 2D-NMR spectroscopic experiments *viz.* HSQC for assignments of *C. caesia* metabolites.**

**Suppl.Fig. S4: 2D-NMR spectroscopic experiments *viz.* HMBC for assignments of *C. caesia* metabolites.**


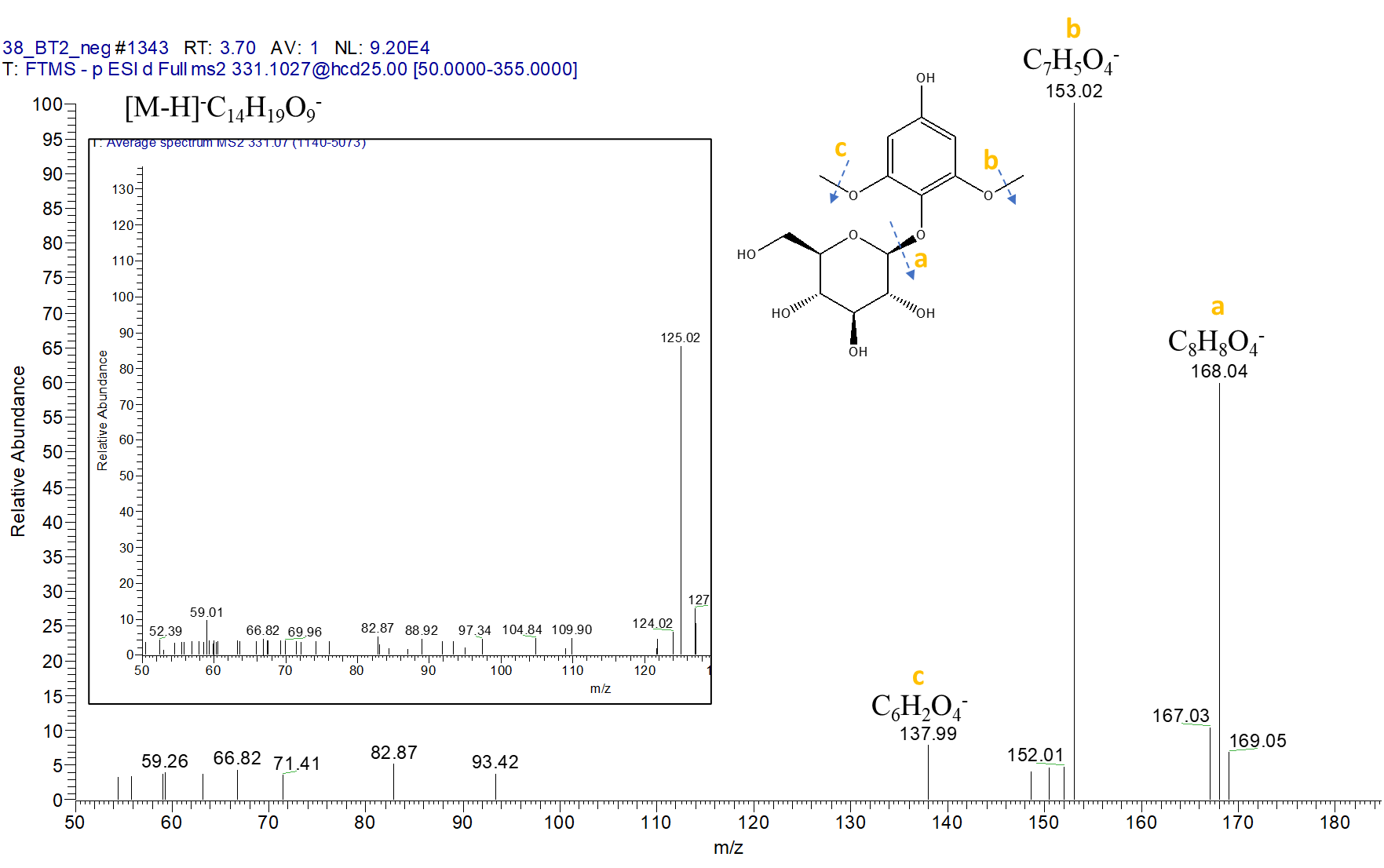


**Suppl.Fig. S5: Tandem MS of dimethoxy-hydroquinone-O-hexoside (peak 16) detected in *C. caesia via*** **UPLC/HR-MS/MS analysis in negative ionization mode**


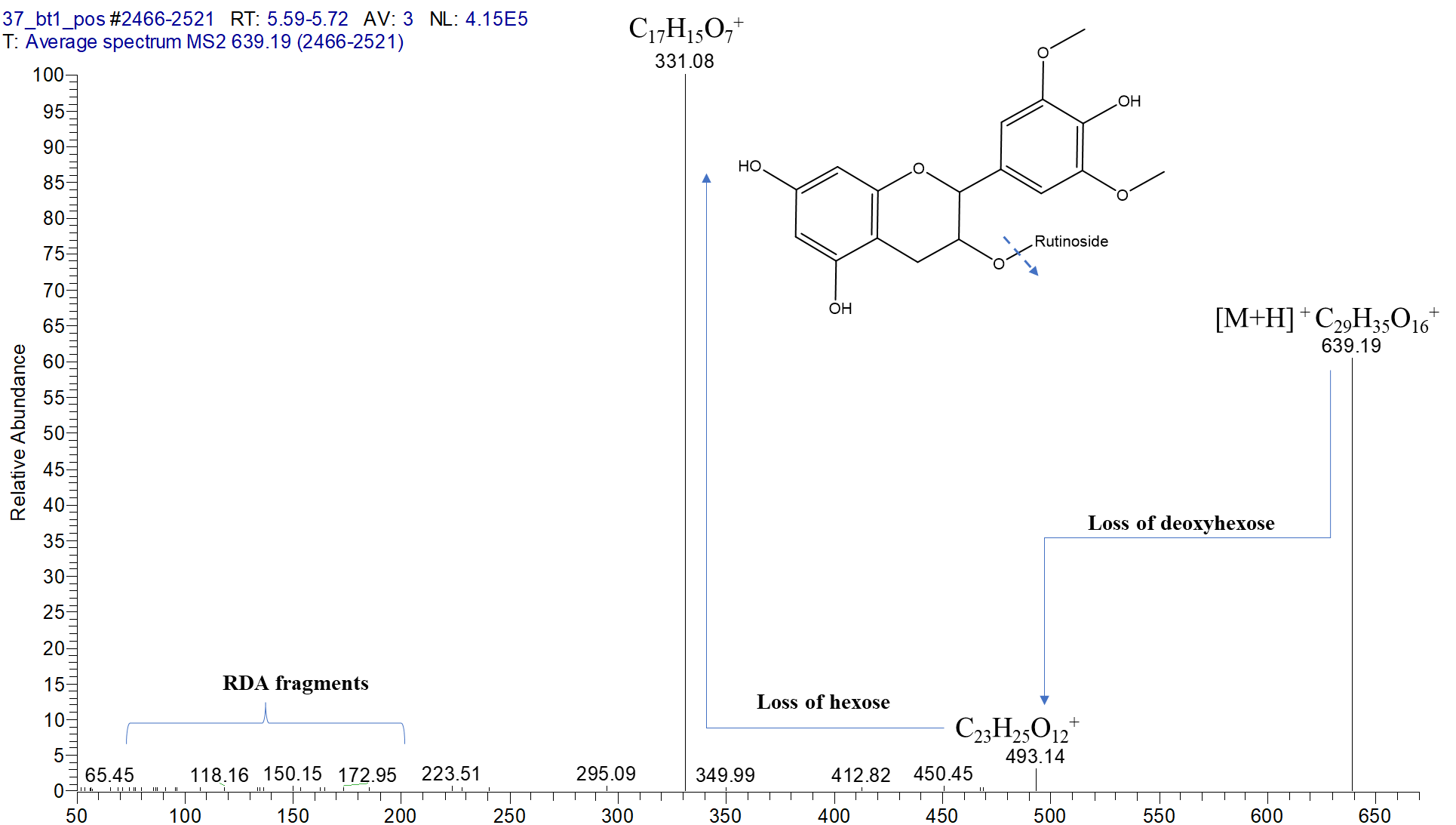


**Suppl.Fig. S6: Tandem MS of malvidin-*O*-rutinoside (peak 23) detected in *C. caesia via*** **UPLC/HR-MS/MS analysis in positive ionization mode**


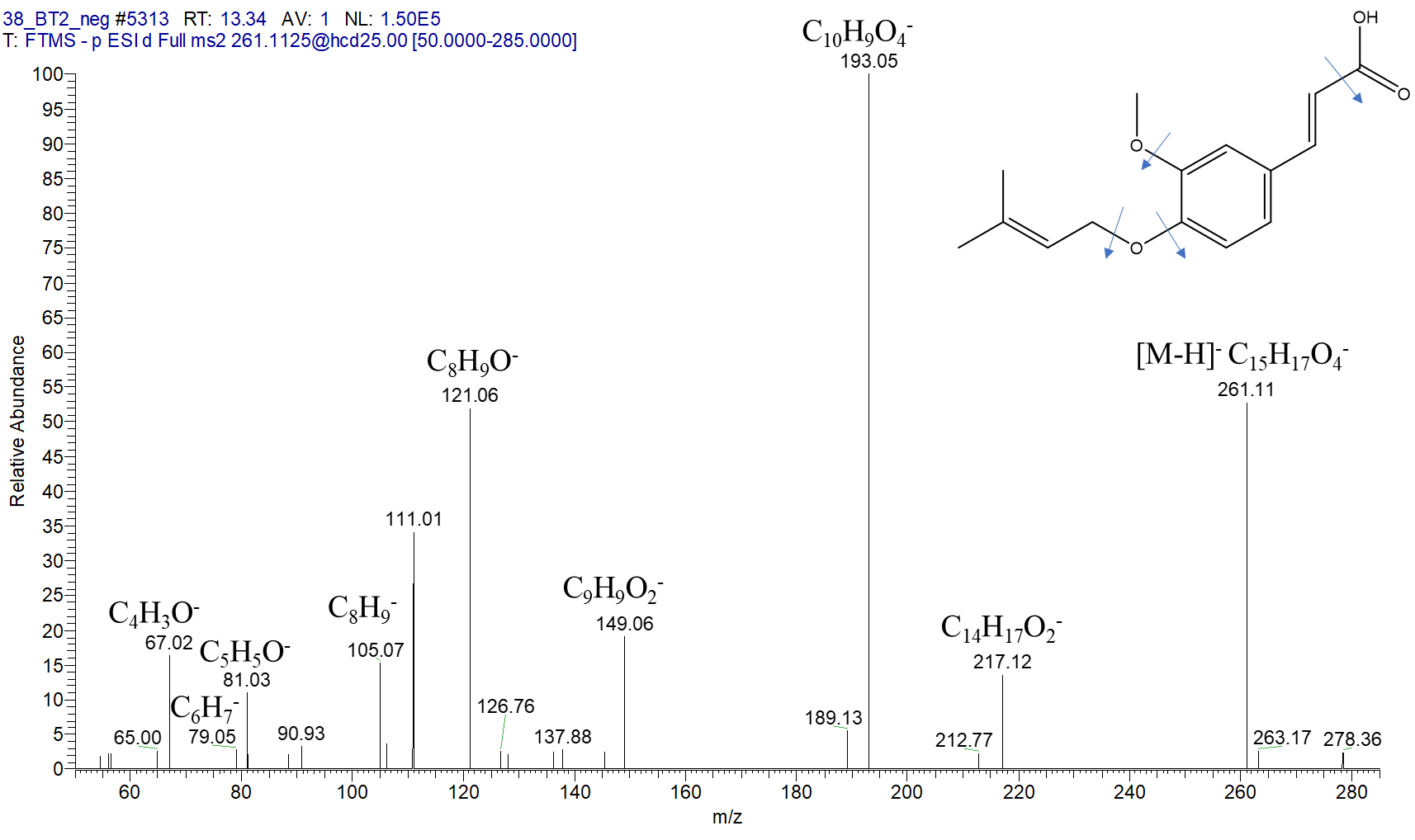


**Suppl.Fig. S7: Tandem MS of methylbutenoxy-ferulic acid (peak 32) detected in *C. caesia via*** **UPLC/HR-MS/MS analysis in negative ionization mode**

**
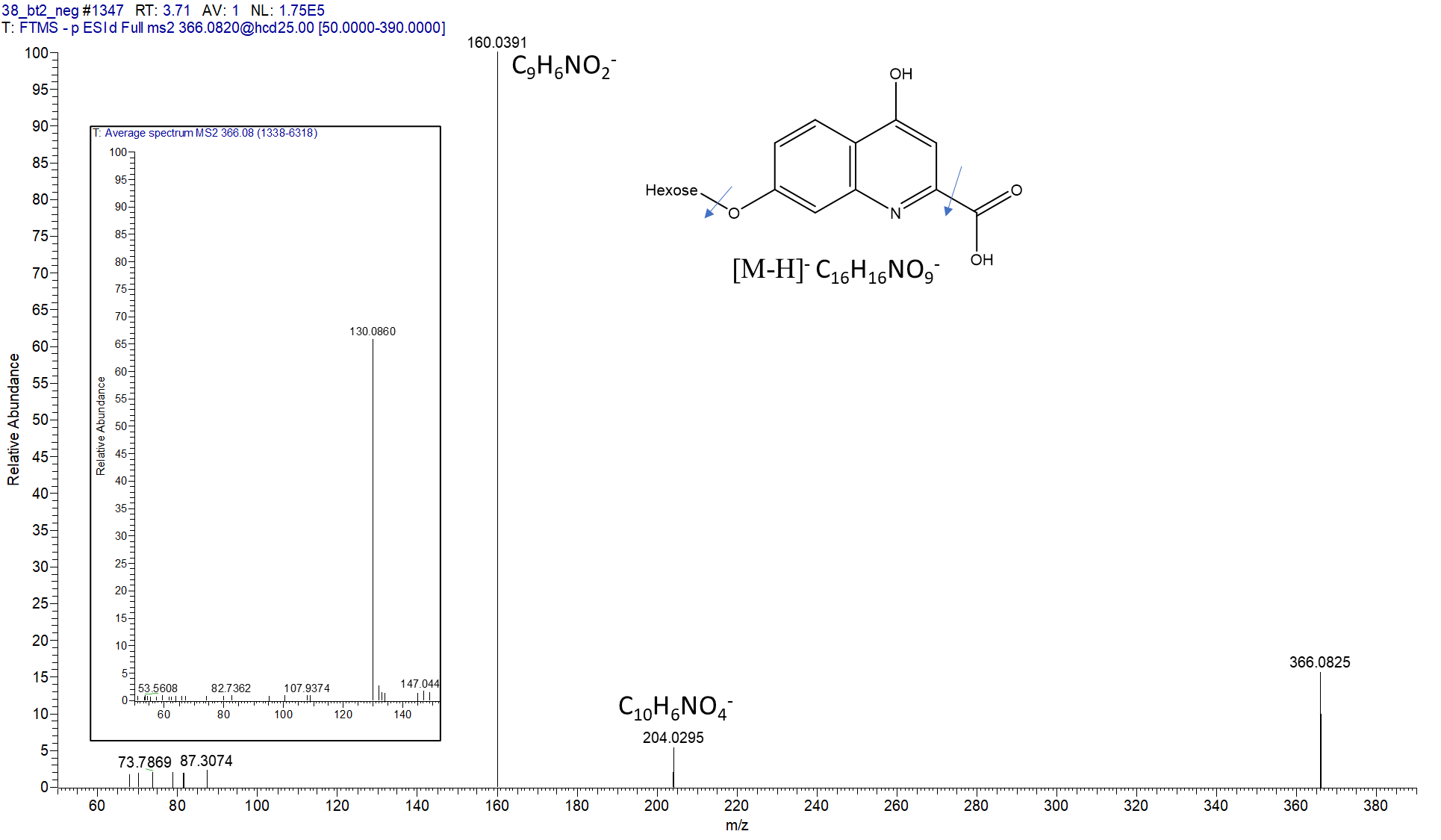
**

**Suppl.Fig. S8: Tandem MS of dihydroxyquinoline carboxylic acid-*O*-hexoside (peak 33) detected in *C. caesia via*** **UPLC/HR-MS/MS analysis in negative ionization mode**

**
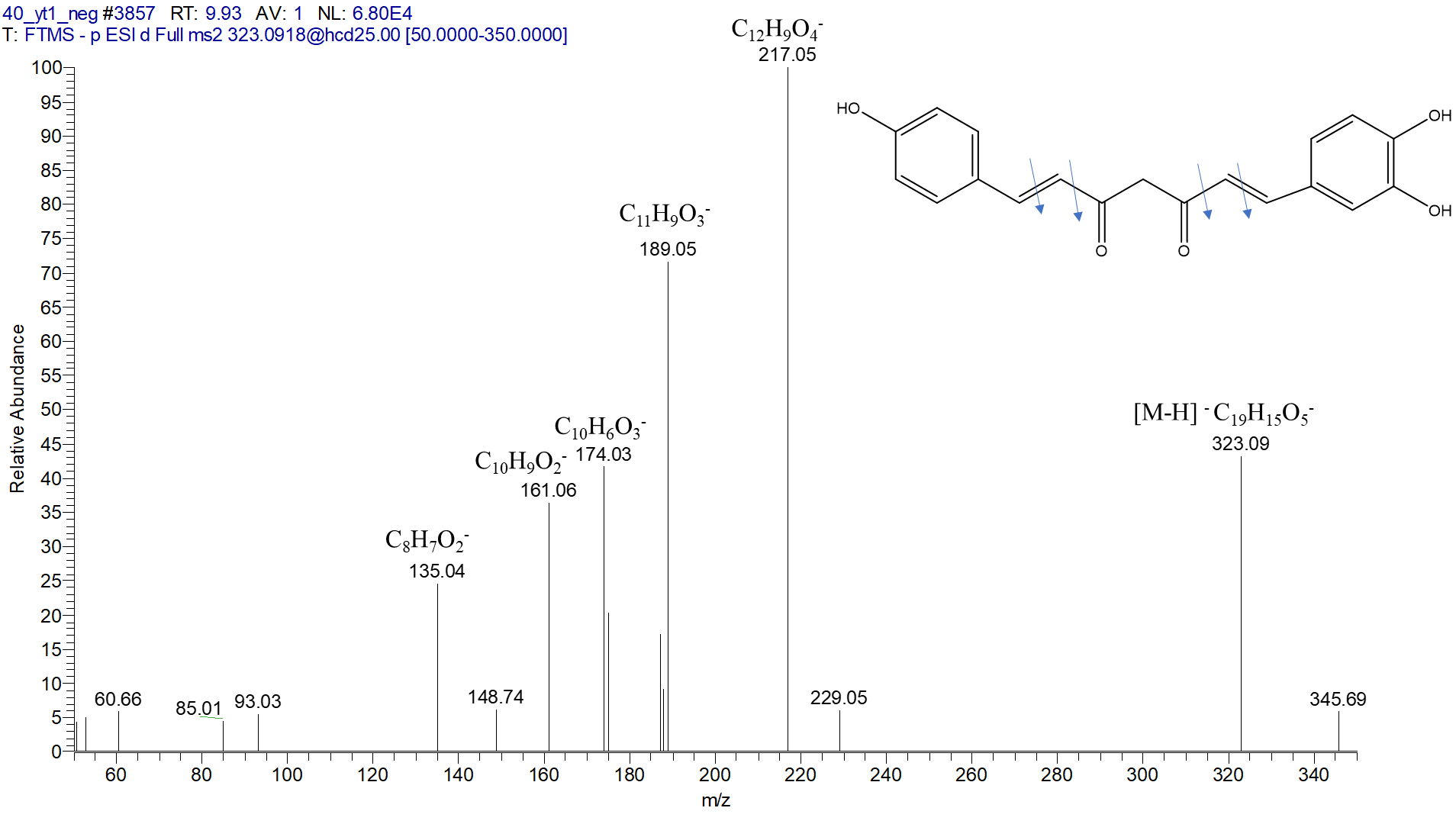
**

**Suppl.Fig. S9: Tandem MS of (hydroxyphenyl)-(dihydroxyphenyl)-heptadiene-dione (peak 38) detected in *C. longa via*** **UPLC/HR-MS/MS analysis in negative ionization mode**

**
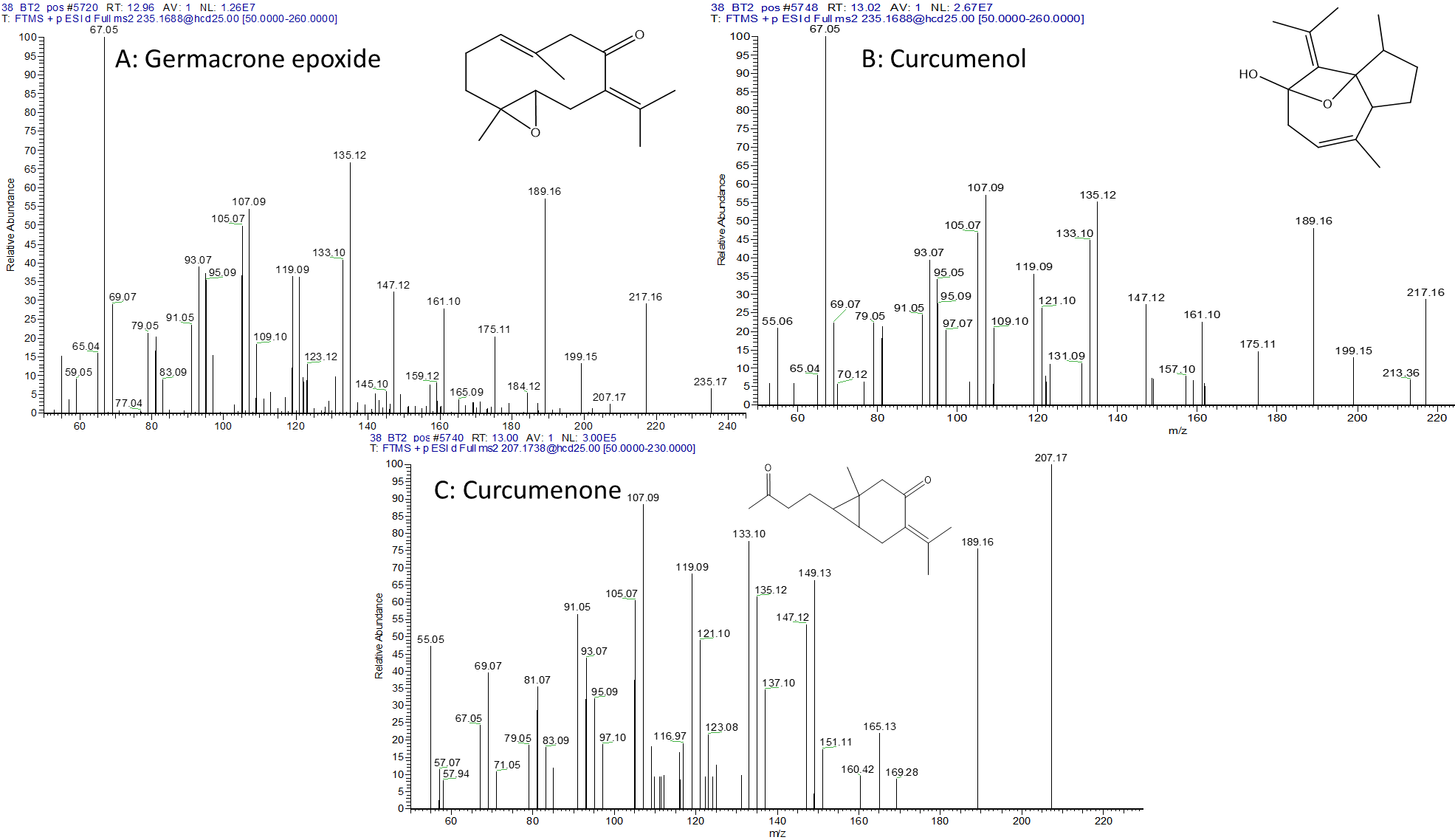
**

**Suppl.Fig. S10: Tandem MS of 3 sesquiterpenes belonging to germacrene (A), guainane (B) and carabrane (C) subtypes having the same molecular ion and elemental composition but distinguished by fragmentation pattern as explained in text (section 3.2.6.)**

**
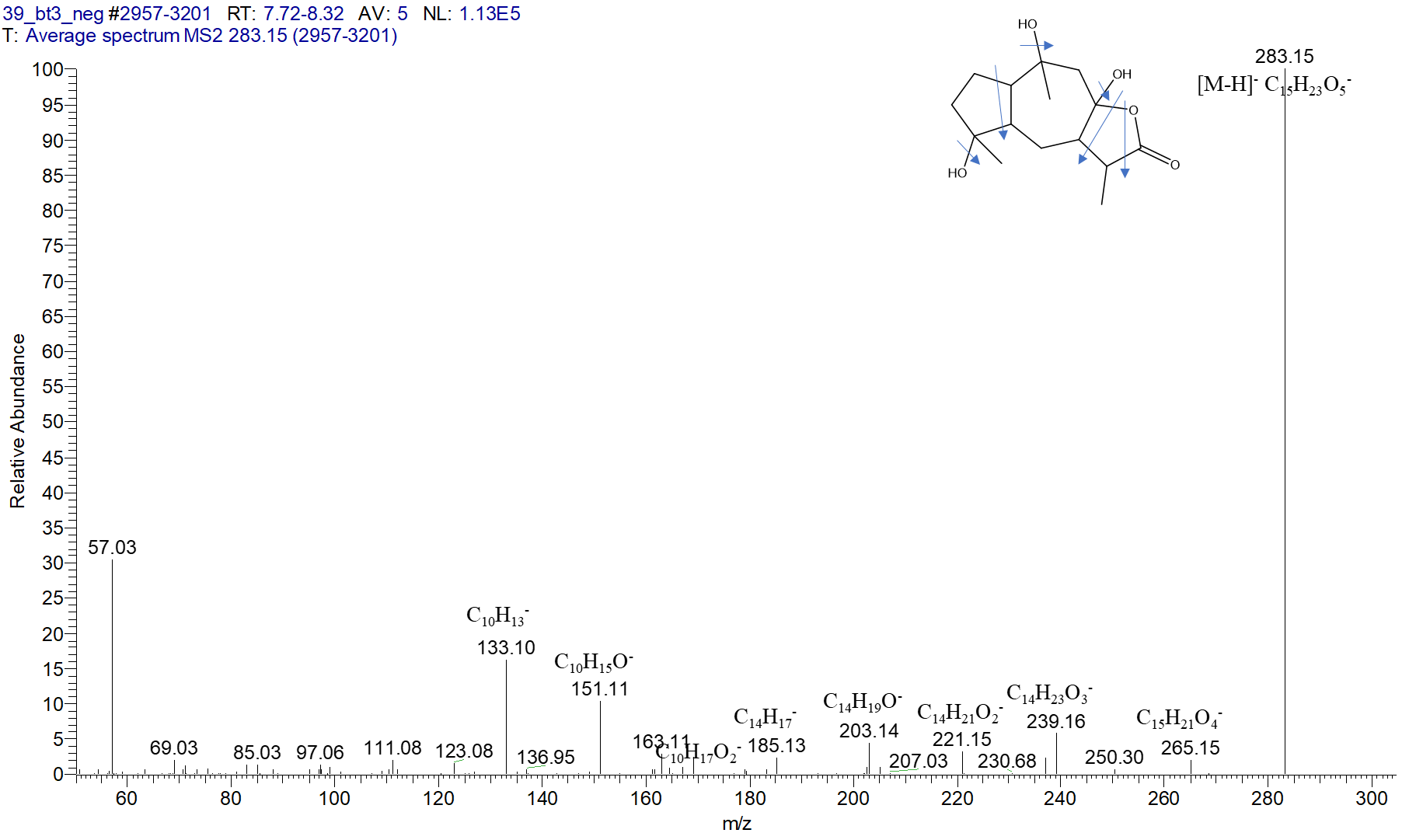
**

**Suppl.Fig. S11: Tandem MS of dihydrozedoarolide B (peak 69) detected in *C. caesia via*** **UPLC/HR-MS/MS analysis in negative ionization mode**


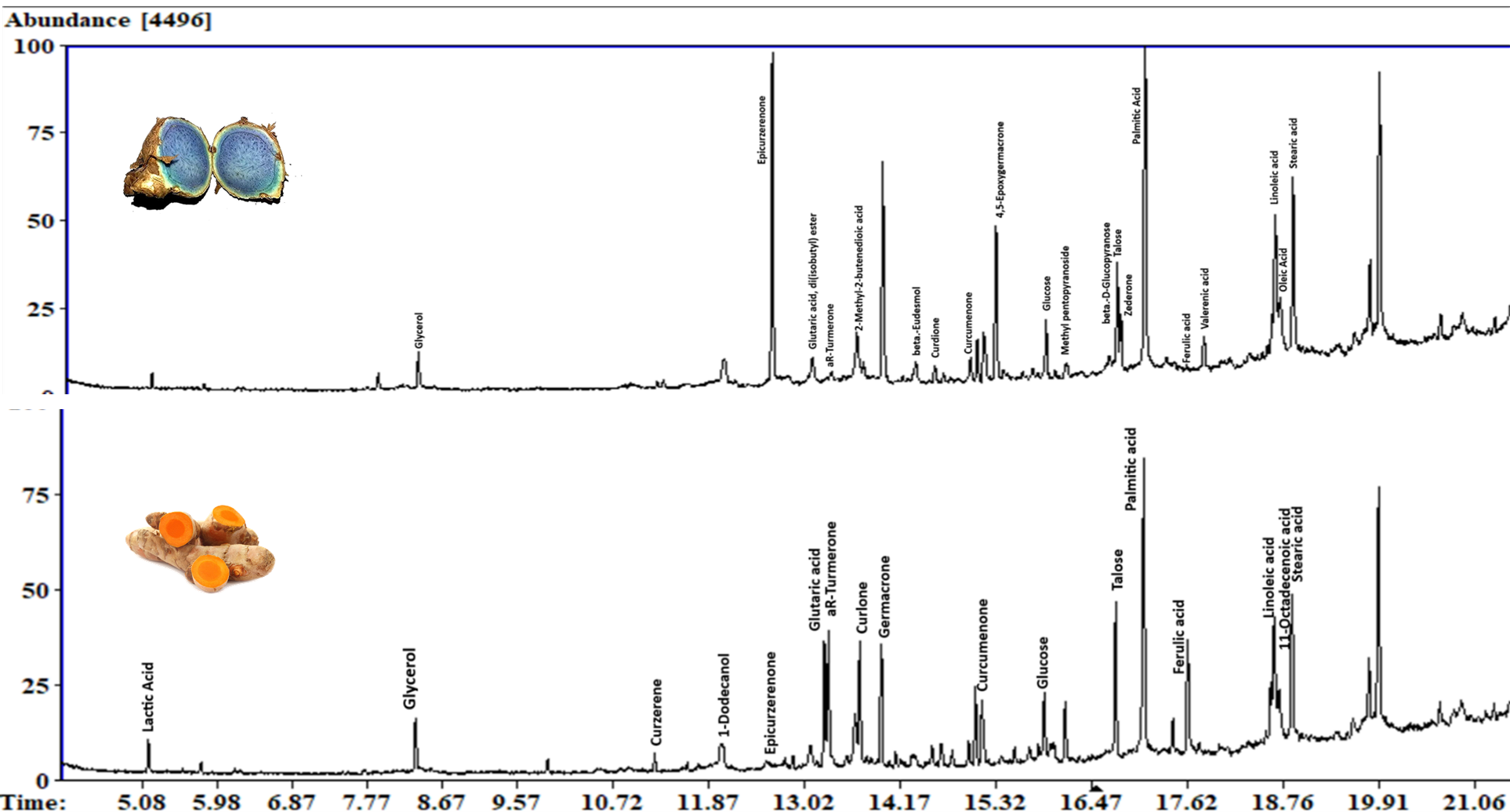


**Suppl.Fig. S12: GC/MS chromatograms of the silylated metabolites detected in *C. caesia* (blue curcuma) and *C. longa* (yellow curcuma) rhizomes specimens**

**
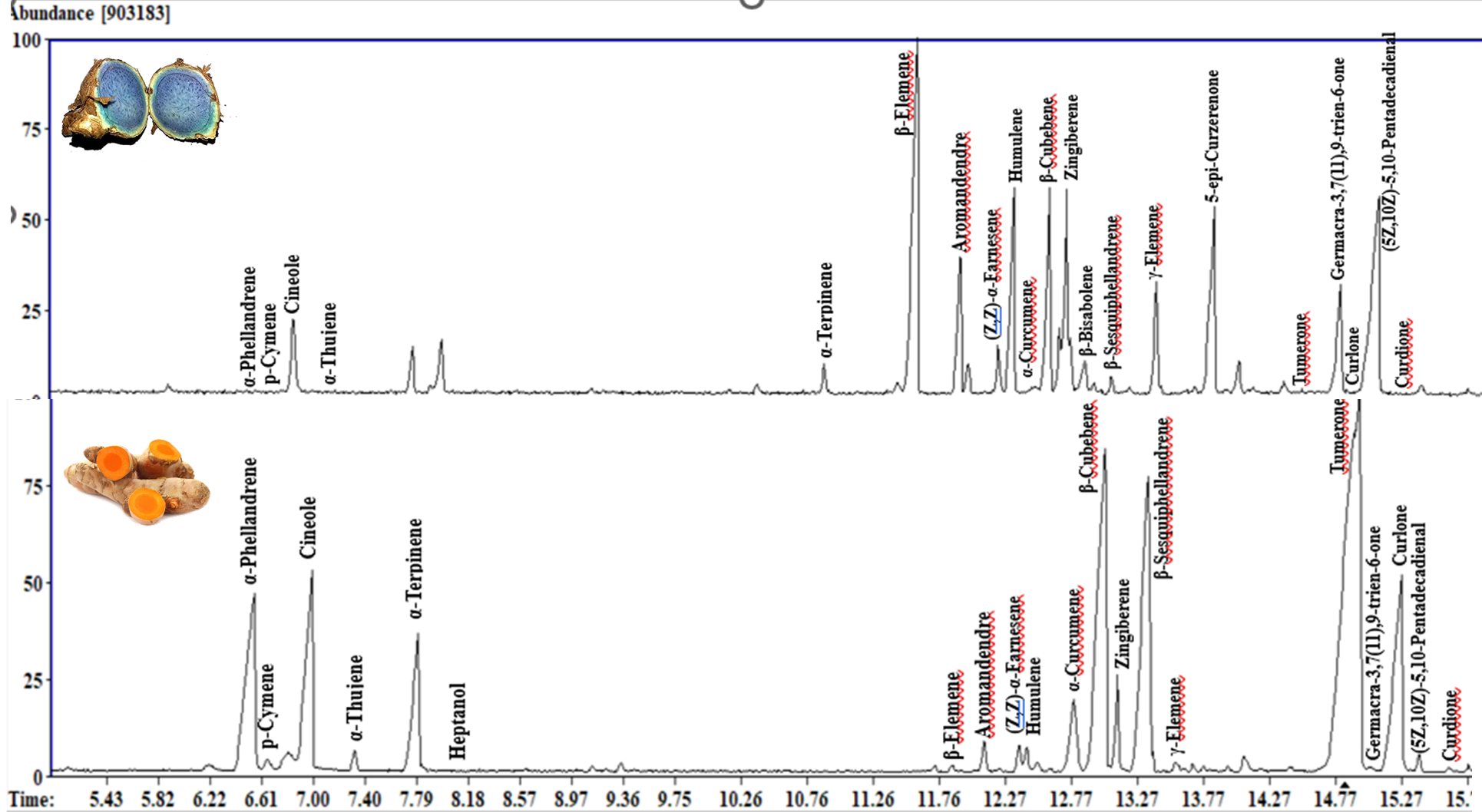
**

**Suppl.Fig. S13: Representative SPME-GC/MS chromatograms of *C. caesia* (blue curcuma) and *C. longa* (yellow curcuma) aroma compounds**

**Suppl.Table 1: ^1^H-NMR quantification of some primary and secondary metabolites**

| **Metabolite** | **Curdione** | **Xanthorrhizol** | **Furanodienone** | **Curcuzederone** | **Curcuminol G** | **Choline** | **Curcumin I** |
| --- | --- | --- | --- | --- | --- | --- | --- |
| **Assignment** | CH_3_-13 | H-12 | H-14 | H-12 | H-15 | CH=CH | H-10, 10` |
| **δ ^1^H (ppm)** | 0.84 | 5.34 | 1.94 | 7.16 | 1,09 | 3.18 | 7.09 |
| **No. of protons** | nd | 1 | 3 | 1 | 3 | 2 | 2 |
| Curcurma caesia (BT) | 0.52 | 0.72 | 0.60 | 0.52 | 0.15 | 0.38 | nd |
| ***Curcurma longa* (YT)** | nd | 1.44 | nd | 0.26 | 0.13 | nd | 0.93 |

**Suppl.Table 2: Relative percentile of volatile constituents detected in *C. caesia* and *C. longa via* SPME-GC/MS analysis**

| **Peak** | **Rt (min.)** | **KI** | **Identification** | ***C. longa*** | ***C. caesia*** |
| --- | --- | --- | --- | --- | --- |
| **Monoterpene hydrocarbon** | | | | | |
| 1 | 6.5 | 869 | α-Phellandrene | 17.18 ± 2.21 | 0.04 ±0.02 |
| 2 | 6.7 | 886 | p-Cymene | 2.27 ±0.27 | 0.05 ± 0.03 |
| 4 | 7.3 | 922 | α-Thujene | 0.19 ±0.04 | 0.01 ±0.00 |
| 5 | 7.7 | 953 | α-Terpinene | 4.64 ±0.60 | 0.02 ±0.01 |
| 7 | 11.07 | 1155 | α-Terpinene | 0.02 ±0.00 | 1.05 ±0.12 |
| 9 | 12.09 | 1229 | Unknown | 0.02 ±0.01 | 0.01 ± 0.01 |
| **Total monoterpene hydrocarbon** | | | | **24.31** | **1.18** |
| **Oxide/ether** | | | | | |
| 3 | 6.9 | 896 | Cineole | 12.64 ±1.38 | 7.83 ± 1.37 |
| **Total oxides** | | | | **12.64** | **7.83** |
| **Alcohol/aldehyde** | | | | | |
| 6 | 8.07 | 974 | Heptanol | 0.04 ±0.03 | 15.09 ±9.36 |
| 25 | 15.3 | 1490 | (5Z,10Z)-5,10-Pentadecadienal | 0.01 ±0.01 | 0.10 ± 0.09 |
| **Total alcohol/aldehyde** | | | | **0.05** | **15.19** |
| **Sesquiterpene hydrocarbon** | | | | | |
| 8 | 11.7 | 1206 | β-Elemene | 0.06 ±0.06 | 18.64 ±0.86 |
| 10 | 12.1 | 1232 | Aromandendrene | 0.79 ±0.13 | 6.57 ±1.14 |
| 11 | 12.3 | 1249 | Unknown | 0.01 ± 0.01 | 0.07 ± 0.06 |
| 12 | 12.4 | 1255 | (Z,Z)-α-Farnesene | 2.02 ±0.53 | 2.71 ±0.77 |
| 13 | 12.5 | 1264 | Humulene | 0.10 ±0.10 | 10.18 ±1.19 |
| 14 | 12.7 | 1280 | α-Curcumene | 0.01 ±0.01 | 0.03 ±0.03 |
| 15 | 12.8 | 1285 | β-Cubebene | 3.71 ±0.70 | 7.56 ±0.45 |
| 16 | 12.9 | 1296 | Curzerene | 0.30 ±0.01 | 0.05 ±0.00 |
| 17 | 12.9 | 1297 | Zingiberene | 1.40 ±0.32 | 12.18 ±2.67 |
| 18 | 13.08 | 1304 | β-Bisabolene | 0.30 ±0.03 | 0.09 ±0.08 |
| 19 | 13.30 | 1322 | β-Sesquiphellandrene | 20.82 ±2.11 | 0.78 ±0.06 |
| 20 | 13.6 | 1353 | γ-Elemene | 0.20 ±0.10 | 4.01 ± 0.43 |
| **Total sesquiterpene hydrocarbon** | | | | **29.17** | **62.79** |
| **Ketone** | | | | | |
| 21 | 14.1 | 1389 | 5-epi-Curzerenone | 0.15 ± 0.11 | 9.50 ±3.78 |
| 22 | 14.8 | 1451 | Turmerone | 33.47 ± 0.77 | 0.01±0.00 |
| 23 | 15.1 | 1471 | Germacra-3,7(11),9-trien-6-one, | 0.08 ± 0.13 | 3.33±2.89 |
| 24 | 15.1 | 1477 | Curlone | 0.09 ± 0.05 | 0.08±0.07 |
| 26 | 15.4 | 1495 | Curdione | 0.01 ± 0.01 | 0.02±0.03 |
| **Total Ketones** | | | | **33.80** | **12.95** |
